# Hydrodeoxygenation of water-insoluble bio-oil to alkanes using a highly dispersed Pd–Mo catalyst

**DOI:** 10.1038/s41467-017-00596-3

**Published:** 2017-09-19

**Authors:** Haohong Duan, Juncai Dong, Xianrui Gu, Yung-Kang Peng, Wenxing Chen, Titipong Issariyakul, William K. Myers, Meng-Jung Li, Ni Yi, Alexander F. R. Kilpatrick, Yu Wang, Xusheng Zheng, Shufang Ji, Qian Wang, Junting Feng, Dongliang Chen, Yadong Li, Jean-Charles Buffet, Haichao Liu, Shik Chi Edman Tsang, Dermot O’Hare

**Affiliations:** 1 Chemistry Research Laboratory, Department of Chemistry, University of Oxford, 12 Mansfield Road, Oxford, OX1 3TA UK; 2 The Wolfson Catalysis Centre, Department of Chemistry, University of Oxford, Oxford, OX1 3QR UK; 3 Beijing Synchrotron Radiation Facility, Institute of High Energy Physics, Chinese Academy of Sciences, 19B Yuquan Road, Shijingshan District, Beijing, 100049 China; 4 Beijing National Laboratory for Molecular Sciences, College of Chemistry and Molecular Engineering, Peking University, Beijing, 100871 China; 5 Department of Chemistry and Collaborative Innovation Center for Nanomaterial Science and Engineering, Tsinghua University, 30 Shuangqing Rd, Haidian Qu, Beijing Shi 100084 China; 6 Product & Technology Development Center, SCG Packaging Public Company Limited, 19 Moo 19 Seang-Xuto Road, ThaPha, BanPong, Ratchaburi, 70110 Thailand; 7 Shanghai Synchrotron Radiation Facilities, Shanghai Institute of Applied Physics, Chinese Academy of Science, 239 Zhangheng Road, Pudong New District, Shanghai, 201204 China; 8 National Synchrotron Radiation Laboratory, University of Science and Technology of China, 42 Hezuohua Road, Hefei, Anhui 230029 China; 9 State Key Laboratory of Chemical Resource Engineering, Beijing University of Chemical Technology, 15 Bei San Huan East Road, Beijing, 100029 China

## Abstract

Bio-oil, produced by the destructive distillation of cheap and renewable lignocellulosic biomass, contains high energy density oligomers in the water-insoluble fraction that can be utilized for diesel and valuable fine chemicals productions. Here, we show an efficient hydrodeoxygenation catalyst that combines highly dispersed palladium and ultrafine molybdenum phosphate nanoparticles on silica. Using phenol as a model substrate this catalyst is 100% effective and 97.5% selective for hydrodeoxygenation to cyclohexane under mild conditions in a batch reaction; this catalyst also demonstrates regeneration ability in long-term continuous flow tests. Detailed investigations into the nature of the catalyst show that it combines hydrogenation activity of Pd and high density of both Brønsted and Lewis acid sites; we believe these are key features for efficient catalytic hydrodeoxygenation behavior. Using a wood and bark-derived feedstock, this catalyst performs hydrodeoxygenation of lignin, cellulose, and hemicellulose-derived oligomers into liquid alkanes with high efficiency and yield.

## Abstract (web summary)

Bio-oil is a potential major source of renewable fuels and chemicals. Here, the authors report a palladium-molybdenum mixed catalyst for the selective hydrodeoxygenation of water-insoluble bio-oil to mixtures of alkanes with high carbon yield.

## Introduction

Decreasing petroleum deposits combined with conversely climbing global demand and environmental concern makes it critical to develop fuel and chemical production processes based on renewable resources^1^. Lignocellulosic biomass, including cellulose, hemicellulose, and lignin, is the most abundantly available and cost-effective carbon reservoir that can be used to produce renewable fuel and high added-value chemicals^2^. Compared with biomass fractionation, which requires extensive pre-treatment and following hydrolysis, flash pyrolysis is a cheaper process for the production of upgradable bio-oil from lignocellulose, and offers potential for lignin utilization^3, 4^. However, the bio-oil also contains high oxygen content (up to 60 wt%) and necessitates further catalytic upgrading, preferably via hydrodeoxygenation (HDO), before use in hydrocarbon fuel or chemical production^5^. The water soluble fraction of bio-oil mainly involves carbohydrates that could be catalytically converted into hydroxymethylfurfural^6, 7^, levulinic acid^8^, and monofunctional hydrocarbons^9, 10^; these platform chemicals can be further converted to hydrocarbons via HDO. By comparison, the water-insoluble bio-oil (WIBO) often contains a large variety of products derived from lignin, cellulose, and hemicellulose in the form of oligomers with molecular weight up to 5000 Da^5^. The complexity of WIBO composition and the chemical inertness of the oligomers present therefore pose a great challenge for any HDO catalyst.

In recent years, bifunctional metal-acid catalysts have been developed, showing high efficiency in HDO reaction for hydrocarbon fuel production from lignin^11–17^. Lercher and co-workers have made important contributions on the design of an efficient combination of metal-catalyzed hydrogenation and acid-catalyzed deoxygenation for highly selective HDO of phenols to cyclohexanes^11, 15^. Both the Dyson and Leitner groups have made impressive progress on the development of high performance catalyst for the HDO of lignin-derived phenols using combinations of metal nanoparticles and acidic ionic liquids^8, 17^. In spite of the growing number of metal-acid combinations, the successful productions of alkanes using these bifunctional metal-acid catalysts have been mostly used for single-component lignin-derived monomers. The conversion of lignin into alkanes has been realized in a rare case via a two-step process, involving catalytic cleavage of the C–O–C bonds and subsequent hydrogenolysis/hydrogenation^18^. Very recently, the production of alkanes, with mass yield up to 28.1 wt%, was reported via HDO from raw lignocellulose containing cellulose, hemicellulose, and lignin by using a Pd/NbOPO_4_ bifunctional catalyst. However, the mass yield of alkyl cyclohexanes converted from lignin was only up to 5.1 wt%^19^. Efficient HDO requires the design and synthesis of a bifunctional catalyst containing active metal and both Brønsted and Lewis acid sites. Recent reports showed that atomically dispersed metal may demonstrate higher activity and efficiency compared with nanoparticles in heterogeneous reactions^20–22^. It is also believed increased Brønsted acid sites favor increasing alkane selectivities^16, 23^, and additional Lewis acid sites may help catalyze the reaction through initial binding of the oxygenated substrates and subsequent cleavage of the C–O linkages^19^.

Herein, we present a highly dispersed Pd–Mo catalyst that encompasses a combination of highly dispersed Pd and ultrafine Mo phosphate nanoparticles on silica (Pd/m–MoO_3_–P_2_O_5_/SiO_2_), in which m represents mixed-valent Mo. In batch reaction, this catalyst exhibits high activity (99%) for the catalytic HDO of phenol to cyclohexane in very mild conditions (383 K, 1 MPa). Furthermore, it shows good re-usability in continuous flow reaction conditions for 419 h. Characterization studies show that the efficient HDO ability may originate from the cooperative effects between highly dispersed Pd, high Brønsted and Lewis acid sites. By feeding wood and bark-derived WIBO containing large amounts of lignocellulosic oligomers, the catalyst not only completely converts phenolic monomers to cyclohexanes, but also achieves HDO of lignin, cellulose, and hemicellulose-derived oligomers into cyclohexanes, hexane, and pentane in 13.4, 5.1, and 7.4 wt%, respectively, with total mass yield up to 29.6 wt% and carbon yield up to 46.3%.

## Results

### Catalysts synthesis and characterizations

**Fig. 1:**
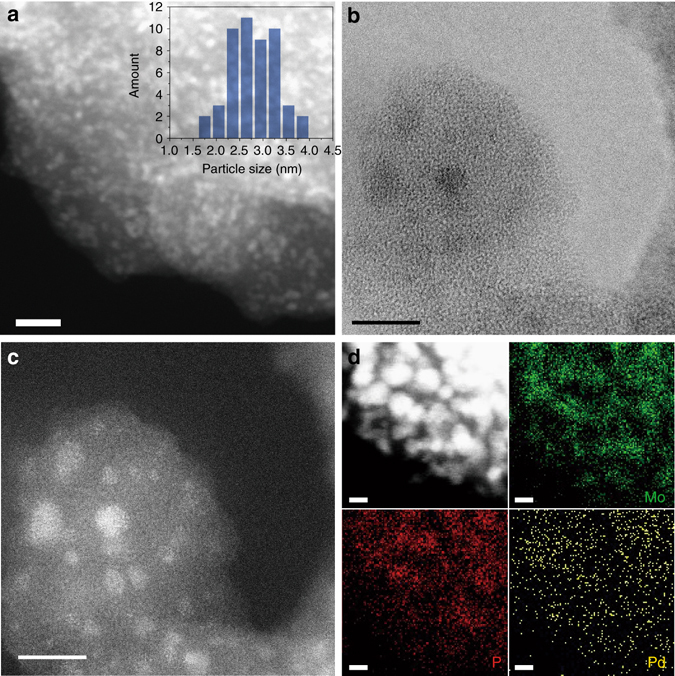
Characterizations of the Pd/m–MoO_3_–P_2_O_5_/SiO_2_ catalyst. **a** HAADF-STEM image of Pd/m–MoO_3_–P_2_O_5_/SiO_2_. *Inset* shows the size distribution of the nanoparticles. *Scale bar* equals 20 nm. **b** Aberration-corrected ABF scanning transmission electron microscope image and **c** corresponding HAADF-STEM image of Pd/m–MoO_3_–P_2_O_5_/SiO_2_. *Scale bars* equal 5 nm. **d** STEM-EDS elemental mapping results for Pd/m–MoO_3_–P_2_O_5_/SiO_2_, showing a homogeneous distribution of Mo and P elements within nanoparticles and highly dispersed Pd. *Scale bar* equals 3 nm

The solid catalyst, Pd/m–MoO_3_–P_2_O_5_/SiO_2_, was prepared using a wet-impregnation method (see “Methods” for preparation). Transmission electron microscopy (TEM) image of the Pd/m–MoO_3_–P_2_O_5_/SiO_2_ (Supplementary Fig. 1) shows ultrafine, electron-rich m–MoO_3_–P_2_O_5_ nanoparticles supported on an amorphous SiO_2_ support. A high-angle annular dark-field scanning transmission electron microscopy (HAADF-STEM) image (Fig. 1a) shows these ultrafine m–MoO_3_–P_2_O_5_ nanoparticles with high coverage and size of 2.8 ± 1.0 nm (*inset*, Fig. 1a). Inductively coupled plasma mass spectrometry (ICP-AES) analysis showed a Mo:P molar ratio of ca. 1:1 (Supplementary Table 1), which is consistent with Energy Dispersive X-ray spectroscopy (EDXS) data (Supplementary Fig. 2). Aberration-corrected annular bright-field (ABF) scanning transmission electron microscope image (Fig. 1b) and corresponding HAADF-STEM image (Fig. 1c) clearly show that the m–MoO_3_–P_2_O_5_ nanoparticles do not form a crystalline lattice. Together with powder X-ray diffractogram (PXRD) data (Supplementary Fig. 3), these results suggest that the m–MoO_3_–P_2_O_5_ nanoparticles are crystallographically disordered. Elemental mapping revealed that Mo and P are well dispersed throughout individual m–MoO_3_–P_2_O_5_ nanoparticles. The structure of the m–MoO_3_–P_2_O_5_ nanoparticles was further investigated with the help of multinuclear solid-state nuclear magnetic resonance (NMR) spectroscopy. The ^31^P solid-state magic angle spinning (MAS) NMR spectrum of a sample of the Pd/m–MoO_3_–P_2_O_5_/SiO_2_ exhibits a single resonance centered at *δ* = –5.5 ppm (Supplementary Fig. 4), the ^31^P chemical shift matches that of a MoO_3_–P_2_O_5_ glass with Mo:P molar ratio ca. 1^24, 25^. A strong absorption band at ca. 966 cm^−1^ in the Raman spectrum (Supplementary Fig. 5) can be assigned to a terminal *ν*(Mo=O) in a MoO_6_ octahedra^25^. The data suggest that this glassy structure consists of chains of inter-connected PO_4_ tetrahedra and MoO_6_ octahedra linked via Mo–O–P bridges.

### X-ray absorption fine structure analysis

**Fig. 2:**
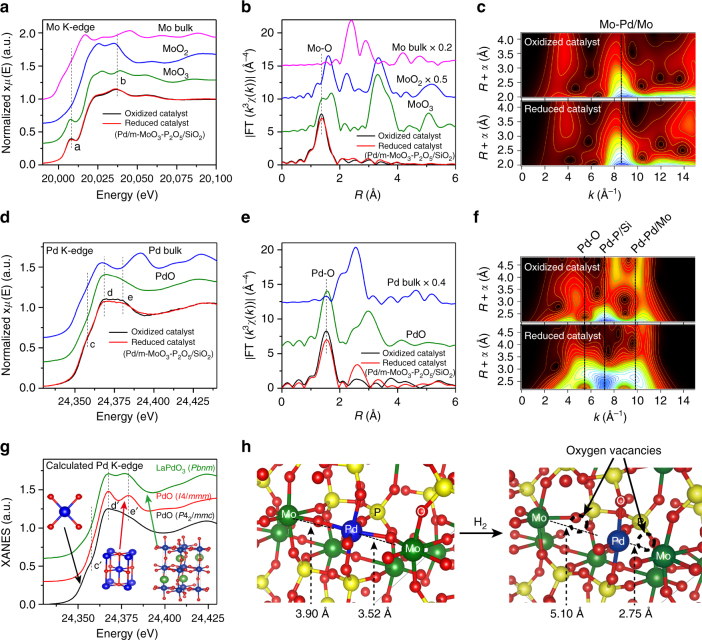
XAFS characterization of the local coordination of Mo/Pd atoms in Pd/m–MoO_3_–P_2_O_5_/SiO_2_ catalyst. **a** The experimental Mo K-edge XANES spectra and **b** EXAFS Fourier transforms of the reduced catalyst, oxidized catalyst, and references. **c** Comparison of the WTs for the *k* ^3^-weighted Mo K-edge EXAFS signals for the high coordination shells in oxidized catalyst and reduced catalyst. **d** The experimental Pd K-edge XANES spectra and **e** EXAFS Fourier transforms of reduced catalyst, oxidized catalyst, and references. **f** Comparison of the WTs for the *k* ^3^-weighted Pd K-edge EXAFS signals for the high coordination shells in oxidized catalyst and reduced catalyst. **g** The theoretical XANES spectra calculated with the depicted square planer (PdO-*P*4_2_/*mmc*) and distorted octahedral (PdO-*I*4/*mmm* and LaPdO_3_-*Pbnm*) structures. **h** Schematic representation of structural modulation due to the production of oxygen vacancies in the oxygen network by H_2_ reduction. The *vertical dashed lines* are drawn to guide the eye

In addition to Mo and P elements, ICP-AES (Supplementary Table 1) and EDXS (Supplementary Fig. 2) data also show the presence of Pd in Pd/m–MoO_3_–P_2_O_5_/SiO_2_ (0.48 wt% from ICP-AES). The Pd signal is highly dispersed according to elemental mapping studies (Fig. 1d), and that the absence of Bragg diffraction of Pd from PXRD (Supplementary Fig. 3), these suggest Pd is presented in very small size. Due to the similar atomic number of Pd and Mo, it is difficult to distinguish Pd using the intensity information in the HAADF-STEM image. To understand the coordination environment and chemical states of Pd in Pd/m–MoO_3_–P_2_O_5_/SiO_2_, X-ray absorption near-edge structure (XANES) and extended X-ray absorption fine structure (EXAFS) spectroscopies were carried out. For clear comparison, Pd/m–MoO_3_–P_2_O_5_/SiO_2_ is labeled as reduced catalyst, and corresponding H_2_ treating precursor is labeled as oxidized catalyst. The Mo K-edge XANES spectrum for reduced catalyst shows a notable shoulder peak (denoted as a) in the pre-edge region (Fig. 2a) as MoO_3_, suggesting the formation of strongly distorted MoO_6_ octahedra having Mo=O bond^26^, in good agreement with the Raman results. The average valence of the Mo ions in the reduced catalyst is estimated to be ca. 5.5 using the linear relation between the Mo oxidation state and the energy position of feature b^27^, indicating the possible existence of mixed-valent Mo^5/6+^ oxide states. The Pd K-edge XANES spectrum is found to exhibit two well-defined features including a shoulder peak c and a doublet peak (d and e) at the *white line* (Fig. 2d); our XANES simulation reveals that the shoulder peak c can be considered to be the fingerprint of Pd^2+^ in square planar coordination, while the intensity of feature e is higher for Pd ions in distorted octahedral coordination than for the square planar coordination (Fig. 2g), consistent with XANES results in the literature^28^. Therefore, this implies that the Pd is highly dispersed in the framework of m–MoO_3_–P_2_O_5_/SiO_2_ via a combination of square planar and distorted octahedral configurations, with an averaged oxidation state between Pd^2+^ and Pd^3+^. This is in agreement with recently reported strategy of stabilizing single Pt atoms on a support interface by adopting a proper coordination^22^. The presence of m–MoO_3_–P_2_O_5_/SiO_2_ matrix may offer an ideal interface to stabilize Pd with high dispersion.

The structural change of Pd/m–MoO_3_–P_2_O_5_/SiO_2_ during H_2_ treatment was also investigated. Interestingly, except for the peak intensity decrease for the Pd–O nearest-neighbor (NN) coordination shells, the two higher shell peaks at distances of 2.5 and 3.5 Å. The Pd K-edge EXAFS Fourier transform of reduced catalyst (Pd/m–MoO_3_–P_2_O_5_/SiO_2_) display a dramatic intensity increase and a radial shift to lower *R* direction, as compared to oxidized catalyst (Fig. 2e); similar phenomenon is shown for the Mo K-edge EXAFS results (Fig. 2b). Meanwhile, complementary wavelet transform (WT) EXAFS analysis^29^ reveals strong WT maxima at about 7.0 and 9.5 Å^–1^, which can be, respectively, associated with the Pd–P/Si and Pd–Pd/Mo scatterings (see Supplementary Fig. 6), at a distance of around 2.55 Å surrounding Pd atoms in reduced catalyst, in marked contrast to the WT maxima distribution in oxidized catalyst (Fig. 2f); slight change is observed for the Mo K-edge WT (Fig. 2c). Those results suggest that more oxygen vacancies are produced by H_2_ reduction in the oxide network of reduced catalyst, which may cause the collapse of the PdO_6_ octahedra by breaking the bridging oxygen atoms with the neighbor polyhedrons. A least-squares curve-fitting analysis was carried out for the multiple coordination shells of Mo and Pd (Supplementary Figs. 7–10 and Supplementary Tables 2 and 3). The coordination numbers of the Pd–O bonding in the first NN coordination sphere for reduced catalyst (oxidized catalyst) is estimated to be 4.9 (5.6) at a distance of 2.02 (2.03) Å, evidencing the transformation of the Pd–O bonding from a distorted octahedral to a square planer coordination. Simultaneously, the number of Pd/Mo atoms in the second NN coordination sphere of Pd in the reduced catalyst is estimated to be 1.5 at a distance of 2.80 Å, much larger than that of 0.4 in the oxidized catalyst, strongly signaling the presence of Pd–Pd/Mo atoms. By EXAFS analysis it is difficult to distinguish Mo from Pd; the data suggest a probability of both Pd–Mo and Pd–Pd neighbors after reduction, as schematically shown in Fig. 2h using density functional theory (DFT) calculation﻿s. The data do allow us to conclude there is an absence of large Pd particles, implying a high dispersion for the Pd atoms (further discussions are shown in Supplementary Note 1 and Supplementary Figs. 11–13). Thus, XAFS data indicate that there are significant oxygen vacancy sites present in close vicinity of Mo and highly dispersed Pd in Pd/m–MoO_3_–P_2_O_5_/SiO_2_.

### Studies of the activity and stability of the catalyst

**Fig. 3:**
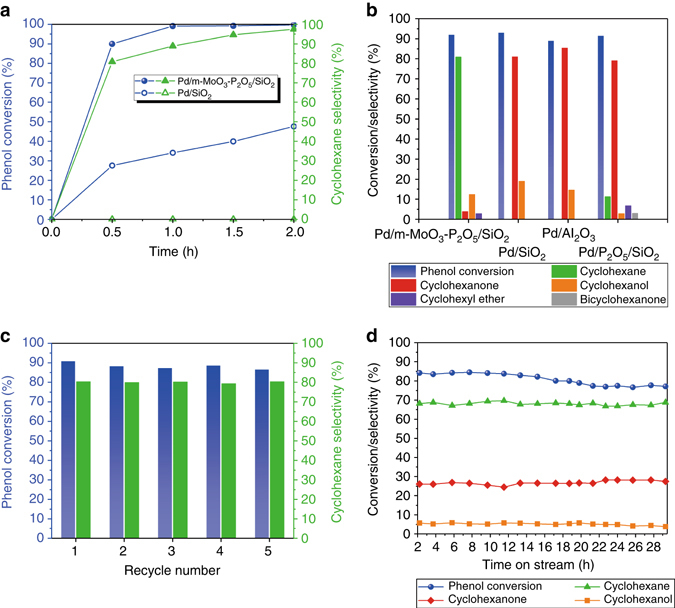
Catalytic performance on HDO of phenol. **a** Time dependence of the phenol conversion and cyclohexanol selectivity using Pd/m–MoO_3_–P_2_O_5_/SiO_2_ and Pd/SiO_2_ catalysts in batch reactions. Reaction conditions: phenol (0.195 mmol), catalyst (including 0.00045 mmol Pd), decalin (7 mL), 383 K, 1 MPa H_2_; reaction mixture stirred at 800 rpm. **b** Comparison of product selectivity of different catalysts at identical conversion level at 383 K, 1 Mpa H_2_ in batch reaction. Reaction conditions: phenol (0.195 mmol), catalyst (including 0.00045 mmol Pd), decalin (7 mL), reaction mixture stirred at 800 rpm. **c** Stability test of Pd/m–MoO_3_–P_2_O_5_/SiO_2_ in batch reaction. Reaction conditions: 383 K, 1 Mpa H_2_, 0.5 h. **d** Long-term stability test on the Pd/m–MoO_3_–P_2_O_5_/SiO_2_ at 433 K, 1 MPa H_2_ with a weight hourly space velocity of 0.17 h^−1^ in a continuous flow reaction

Phenol HDO in a batch reactor was initially chosen as a model reaction to evaluate the catalytic activity of Pd/m–MoO_3_–P_2_O_5_/SiO_2_. The total mass of Pd was kept constant in order to benchmark other catalyst systems (Supplementary Table 1). The reaction process using Pd/m–MoO_3_–P_2_O_5_/SiO_2_ catalyst (Fig. 3a and Supplementary Fig. 14) shows an increase in cyclohexane selectivity and a decrease in cyclohexanol selectivity. In comparison, Pd/SiO_2_ catalyst only delivered hydrogenated products (cyclohexanone and cyclohexanol) and subsequent dehydration was not observed (Supplementary Fig. 14). These results suggest that in the case of Pd/m–MoO_3_–P_2_O_5_/SiO_2_ catalyst, phenol is initially hydrogenated to cyclohexanol catalyzed by Pd, which is followed by dehydration of cyclohexanol catalyzed by m–MoO_3_–P_2_O_5_ and further hydrogenation of cyclohexene to yield cyclohexane catalyzed by Pd. Via a comparison of product selectivity at near identical conversion level (Fig. 3b), the Pd/m–MoO_3_–P_2_O_5_/SiO_2_ catalyst exhibited a much higher cyclohexane selectivity (81%) compared with catalysts without m–MoO_3_–P_2_O_5_, indicating its high dehydration ability is due to the presence of m–MoO_3_–P_2_O_5_. We have compared the performance of Pd/m–MoO_3_–P_2_O_5_/SiO_2_ for the HDO of phenol to a series of state-of-art bifunctional metal-acid catalysts (Supplementary Table 4). Overall Pd/m–MoO_3_–P_2_O_5_/SiO_2_ demonstrates higher phenol conversions, with better cyclohexane selectivity at a lower temperature and lower H_2_ pressure than any of other catalyst systems. The catalyst was re-used and showed no obvious deactivation after five cycles (Fig. 3c), showing its stability under batch reaction conditions. In order to investigate stability under more industrially related conditions, Pd/m–MoO_3_–P_2_O_5_/SiO_2_ was evaluated under continuous flow condition at 433 K, 1 MPa H_2_ with flow rate of 10 cm^3^ (STP)min^–1^, weight hour space velocity (WHSV) = 0.18 h^–1^. The results show that there is some degree of deactivation (phenol conversion from 84.2 to 77.2%) but stable cyclohexane selectivity (from 68.2 to 68.8%) in the 32-h test (Fig. 3d), exhibiting higher cyclohexanol selectivity and stability than those in recent report under similar conditions (cyclohexane yield decrease from 52 to 28% in 4 h)^17^. The spent catalyst shows no obvious change (Supplementary Figs. 15, 16). The Mo and Pd K-edge XANES and EXAFS spectra are also given for the spent catalyst, exhibiting no obvious changes in the local structure around Mo and Pd atoms (Supplementary Fig. 17). The deactivation was mainly due to carbonaceous deposition (Supplementary Fig. 18). To evaluate re-use ability, Pd/m–MoO_3_–P_2_O_5_/SiO_2_ was evaluated under continuous flow conditions (453 K, 1 MPa H_2_ with WHSV of 0.085 h^−1^). As shown in Supplementary Fig. 19, after it was deactivated (phenol conversion from >99 to 69.0%, cyclohexanol selectivity from 98.3 to 90.4%) in 135 h, the spent catalyst was calcined at 673 K for 5 h to remove the carbonaceous deposition and re-used for another two runs. The result shows that the initial performance could be substantially recovered, with phenol conversion of >99% (1st run), 94.5% (2nd run), and 94.0% (3rd run), respectively, and cyclohexane selectivity of 98.3% (1st run), 98.4% (2nd run), and 96.7% (3rd run), respectively, and a similar deactivation trend was observed. This good re-usability ensures a long-term reaction efficiency (maintaining phenol conversion >60% and cyclohexane selectivity >90%) in three runs for over 419 h without obvious catalyst leaching (Supplementary Table 1).

### Studies of the effects of Brønsted and Lewis acidity on reaction

**Fig. 4:**
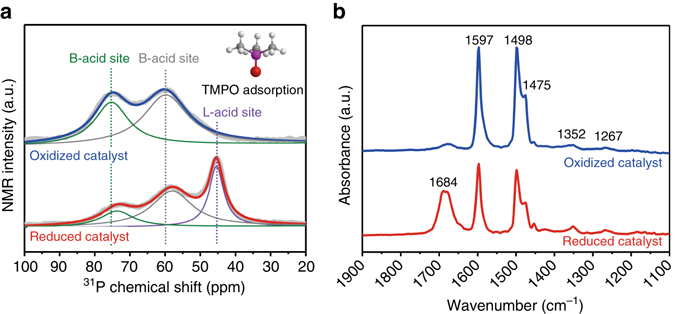
Surface analyses of Pd/m–MoO_3_–P_2_O_5_/SiO_2_ catalyst. **a** Solid-state ^31^P MAS NMR spectra of TMPO-adsorbed on the oxidized catalyst and reduced catalyst (Pd/m–MoO_3_–P_2_O_5_/SiO_2_). *Inset* displayed TMPO molecule. **b** DRIFTS spectra at 1100–1900 cm^−1^ obtained over oxidized catalyst and reduced catalyst (Pd/m–MoO_3_–P_2_O_5_/SiO_2_) in a reaction mixture containing phenol and He at 383 K

^31^P MAS NMR spectrum were recorded following adsorption of the trimethylphosphine oxide (TMPO) probe molecule, giving information about the acid type (Brønsted vs. Lewis), strength and concentration in the materials^30^. The ^31^P MAS NMR spectrum (Fig. 4a) of the TMPO-adsorbed on reduced catalyst (Pd/m–MoO_3_–P_2_O_5_/SiO_2_) exhibits two distinct resonances at 58.0 and 73 ppm, both signals are attributed to the protonation of TMPO by surface Brønsted acid sites with exceptionally high density reaching 0.0138 mmol/m^2^ (Supplementary Table 5 and Supplementary Fig. 20), which is 2.6-fold higher than a strong acidic zeolite HZSM-5 (Si to Al of 15)^31^. The reduced catalyst exhibits another new resonance at 45 ppm, which was not observed in oxidized catalyst and could be attributed to Lewis acid with density of 0.0075 mmol/m^2^. A series of catalysts with different Brønsted acid sites and Lewis acid sites were prepared by the same procedure as the Pd/m–MoO_3_–P_2_O_5_/SiO_2_ catalyst by varying the molar ratio of P:Mo (Supplementary Table 5). The existence of the Lewis acid site is observed only on catalysts containing Mo species, thus it is most likely linked to oxygen vacancy associated with Mo centers^32^, which is well consistent with abovementioned XAFS analysis. The formation of oxygen vacancy is observed by temperature programmed reduction (TPR, Supplementary Fig. 21), and could be linked with the reduction of surface oxidation state evidenced by multi-frequency electron paramagnetic resonance (EPR, see Methods, Supplementary Figs. 22–24) and by solid-state magnetic susceptibility measurements (Supplementary Fig. 25).

We believe that the excellent performance of Pd/m–MoO_3_–P_2_O_5_/SiO_2_ for HDO may be attributed to cooperative effects between highly dispersed Pd, strong Brønsted acid sites, and Lewis acid sites. The highly dispersed Pd allows for higher hydrogenation activity (Fig. 3a) and enhanced metal atom efficiency compared with Pd/SiO_2_ (Supplementary Fig. 14), by increasing the number of exposed surface atoms by using very low Pd loading amount of 0.48 wt%. The catalysts with different Brønsted acid sites (Supplementary Table 5) were compared in the dehydration of cyclohexanol (Supplementary Table 6), which is the most important intermediate in the HDO of phenol. Since most of the samples show comparable Brønsted acid strength, respectively, revealed by resonance position of ^31^P MAS NMR spectrum (except Pd/P_2_O_5_/SiO_2_ (P:Mo = 1:0) which shows weaker Brønsted acid strength), one can look into the correlation between Brønsted acid and dehydration activity. The result shows that only samples containing Brønsted acid sites have activity, suggesting the dehydration is a Brønsted acid-catalyzed process. Specifically, Pd/m–MoO_3_–P_2_O_5_/SiO_2_ (P:Mo = 1:1, the reduced catalyst shown in the main work) with the most Brønsted acid sites is a more active dehydration catalyst, and also Pd/m–MoO_3_–P_2_O_5_/SiO_2_ (P:Mo = 2:1) with higher Brønsted acid sites compared with Pd/MoO_3_/SiO_2_ (P:Mo = 0:1) has higher dehydration activity (conversion: 25.2 vs. 19.8%), which are consistent with the correlation between the Brønsted acid sites and the dehydration activity^16, 23^. It is also observed that Pd/P_2_O_5_/SiO_2_ has lower dehydration activity compared with Pd/MoO_3_/SiO_2_ (12.8 vs. 19.8%), although its Brønsted acid sites is higher. This could be due to its relatively weaker Brønsted acid strength^13^. The role of Lewis acid site on phenol conversion was further studied by performing diffuse-reflectance infrared Fourier transform spectroscopy (DRIFTS) upon exposure of the oxidized and reduced catalyst (Pd/m–MoO_3_–P_2_O_5_/SiO_2_) to a phenol/He mixture at reaction temperature (383 K). In the measurements, the reduced catalyst was first treated with 5% H_2_/Ar at 383 K before exposed to phenol/He mixture. The DRIFTS spectra of both reduced catalyst and oxidized catalyst (Fig. 4b) show bands at 1597, 1497, 1475, 1352 and 1267 cm^–1^, which correspond to different vibrational modes of phenoxy species^33^. Compared with oxidized catalyst, the reduced catalyst shows more pronounced bands at 1684, 2951 and 2874 cm^–1^ (Fig. 4b, Supplementary Fig. 26), which could be assigned to the *v*(C=O) and *v*(C–H) stretching modes, respectively, of adsorbed 2,4-cyclohexadien-1-one tautomer of phenol^33, 34^. Considering that both the DRIFTS of spectra of oxidized Pd/SiO_2_ and reduced Pd/SiO_2_ do not show obvious bands for 2,4-cyclohexadien-1-one (Supplementary Fig. 27), the formation of this compound is more likely promoted by Lewis acid sites (oxygen vacancy associated with Mo centers) on reduced catalyst via tautomerization of phenol. This compound is an important intermediate in the HDO of phenol supported by Priscillat et al. studies^33^, which could be first hydrogenated to 2-cyclohexen-1-one, then to cyclohexanone and further to cyclohexanol.

### HDO of WIBO

**Fig. 5:**
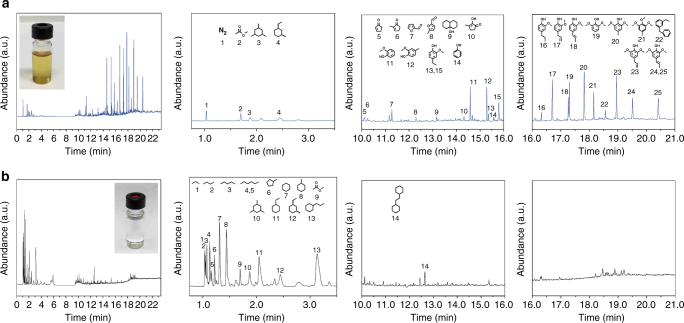
GC-MS analysis before and after upgrading. **a** GC-MS analysis of WIBO dissolved in decalin before upgrading. **b** GC-MS analysis of upgrading product after a batch reaction. Reaction conditions: WIBO (86.2 mg), Pd/m–MoO_3_–P_2_O_5_/SiO_2_ (170.8 mg), decalin (7 mL), 453 K, 1 MPa H_2_, 4 h, stirred at 800 rpm. MS detector was off from 6.2 to 9.5 min, and 11.8 to 11.9 min to block decalin and tetralin signals. The overall GC-MS patterns and three enlarged regions are shown. *Insets* show the images of mixtures before and after upgrading, respectively

Based on the above understanding, efficiency test using Pd/m–MoO_3_–P_2_O_5_/SiO_2_ for upgrading a WIBO derived from fast pyrolysis of grounded eucalyptus wood chip and bark were carried out. This WIBO sample was a dark red, treacle-like, viscous liquid (Supplementary Fig. 28) with a 47.2 wt% carbon content and a 42.8 wt% oxygen content (Supplementary Table 7). The WIBO contains 13 wt% moisture determined by Karl Fisher method, which mainly derived from the feedstock and also the dehydration reactions during flash pyrolysis treatment^35^. The content of the WIBO was studied using gas chromatograph-mass spectrometry (GC-MS) (Fig. 5a and Supplementary Fig. 29). The results show the WIBO contains a series of oxygenated monomers involving phenolic compounds such as guaiacol, syringol, and furans (4.7 wt% of total WIBO mass). High-performance liquid chromatography (HPLC) of the water extract of the WIBO (Supplementary Fig. 30) showed no sugar-type monomers before it was hydrolyzed. The remaining mass can be attributed to lignin and sugar oligomers that are undetectable in both GC-MS and HPLC. Gel permeation chromatography (GPC) analysis (Supplementary Fig. 31) verified the presence of oligomers in the WIBO with molecular weight up to 1000 Da and weight average molecular weight (*M*_w_) of 401. This *M*_w_ value is consistent with those reported for wood pyrolysis oils^36^.

HDO of this WIBO was carried out in a batch reactor at 453 K and 1 MPa H_2_ in the presence of Pd/m–MoO_3_–P_2_O_5_/SiO_2_ catalyst. Total conversion of the oxygenated monomers was achieved after 4 h (Fig. 5b), with the production of 9.4 wt% total mass yield of liquid alkanes involving 0.9 wt% pentane, 1.2 wt% hexane, and 5.6 wt% C_6_–C_9_ cycloalkanes (cyclohexane, methylcyclohexane, ethylcyclohexane, and propylcyclohexane; see Table 1 and Supplementary Fig. 32). Overall a 10.2% carbon yield in the form of cyclohexanes was obtained. This is higher than the maximum carbon yield (5.7%) possible from the lignin-derived monomers present in the WIBO. This indicates that some oligomers in the WIBO were converted into cyclohexanes. The production of pentane and hexane suggests that HDO of cellulose and hemicellulose into alkanes are achieved, which is supported by the earlier literature^19^. The color of the mixture became lighter after the reaction, serving as another qualitative indicator of the HDO efficiency (*inset* in Fig. 5a, b). To further increase the mass yield of liquid alkanes, the reaction was carried out at an increased temperature. At 523 K and 1 MPa H_2_, 29.6 wt% total mass yield and 46.3% total carbon yield of liquid alkanes was achieved after 15 h, the mass yield of cyclohexanes, pentane, and hexane reached 13.4, 7.4 and 5.1 wt%, respectively (see Methods, Table 1, Supplementary Fig. 33). It should be noted that the total carbon yield is comparable to the state-of-art in catalytic performance^19, 37, 38^. GPC analysis (Supplementary Fig. 34) shows the decrease of oligomers. Although reaction mechanisms for HDO of different classes of oxygenates in bio-oil could be different, these results clearly indicate that the catalyst has a high performance for simultaneous HDO of lignin, cellulose, and hemicellulose-derived oligomers into liquid alkanes.

**Table 1:** HDO of water-insoluble wood and bark-derived WIBO over Pd/m–MoO_3_–P_2_O_5_/SiO_2_ catalyst

Products	Mass yield^a^/carbon yield^b^ (wt%/wt%)	
	Entry 1^c^	Entry 2^d^
Pentane	0.9/1.5	7.4/11.4
Hexane	1.2/1.8	5.1/7.9
Methylcyclopentane	0.9/1.4	3.7/5.8
Cyclohexanes	5.6/10.2	13.4/21.2
Total liquid alkanes	9.4/14.8	29.6/46.3
Others^e^	1.2/2.2	6.8/10.2

^a^On the basis of the mass of moisture-free WIBO

^b^On the basis of the mass of carbon in WIBO

^c^Reaction condition: WIBO (86.2 mg), Pd/m–MoO_3_–P_2_O_5_/SiO_2_ (170.83 mg), decalin (7 mL), 453 K, 1 MPa H_2_, 4 h, stirred at 800 rpm

^d^Reaction condition: WIBO (87.2 mg), Pd/MoO_3_–P_2_O_5_/SiO_2_ (170.8 mg), decalin (7 mL), 523 K, 1 MPa H_2_, 15 h, stirred at 800 rpm

^e^Others mainly include C_3_–C_4_ alkanes

## Discussion

We report a highly active HDO catalyst comprising highly dispersed Pd and ultrafine Mo phosphate nanoparticles supported on SiO_2_. This catalyst is 100% effective and is 97.5% selective for HDO of phenol to cyclohexane under mild conditions, showing regeneration ability in long-term continuous flow tests for 419 h with some decrease in catalytic performance after 32 h in a continuous flow reaction. There are cooperative effects between highly dispersed Pd, Brønsted and Lewis acid sites on Pd/m–MoO_3_–P_2_O_5_/SiO_2_, which shows to be important for the efficient catalytic HDO behavior. Using water-insoluble fraction of wood and bark-derived WIBO, the catalyst showed state-of-art performance for the simultaneous HDO of lignin, cellulose, and hemicellulose-derived oligomers into liquid alkanes with total mass yield of 29.6 wt% under mild condition (523 K, 1 MPa H_2_). Further work will include mechanistic investigations for HDOs of different classes of oxygenates in bio-oil to fully understand the high efficiency of this catalyst.

## Methods

### Sample preparation

Pd/m–MoO_3_–P_2_O_5_/SiO_2_ synthesis: (NH_4_)_6_Mo_7_O_24_•4H_2_O (0.021 mmol), (NH_4_)_2_HPO_4_ (0.150 mmol), citric acid (0.30 mmol) were dissolved in 0.3 mL of HCl aqueous solution containing PdCl_2_ (1.5 mg). The mixture was stirred for 1 h and then added dropwise to SiO_2_ (0.12 g). The obtained glue-like sample was dried in an oven at 397 K overnight and calcined at 773 K in a muffle furnace for 5 h at a rising rate of 5 K/min. The calcined sample, named as Pd/MoO_3_–P_2_O_5_/SiO_2_, was reduced in H_2_ at 323 K for 3 h with a flowing rate of 75 cm^3^/min. The calcined sample changed to a greenish-blue color following reduction. This reduced sample was named Pd/m–MoO_3_–P_2_O_5_/SiO_2_. The synthesis of Pd/P_2_O_5_/SiO_2_ was similar as for Pd/m–MoO_3_–P_2_O_5_/SiO_2_, except with no addition of (NH_4_)_6_Mo_7_O_24_•4H_2_O. Pd/SiO_2_ was prepared by dropwise addition of 0.3 mL of prepared PdCl_2_ solution into SiO_2_ under strong stirring. The following operations are the same as the one for Pd/m–MoO_3_–P_2_O_5_/SiO_2_. The fresh catalyst was protected in N_2_ and quickly prepared as samples for further characterizations and catalytic tests.

### High-resolution TEM

HRTEM images were acquired with JEOL JEM-2100F field-emission TEM with an accelerating voltage of 200 kV.

### Aberration-corrected TEM

Aberration-corrected TEM was performed on JEOL 200F TEM operated at 200 keV. The attainable spatial resolution of the microscope is 78 pm with a probe spherical-aberration corrector. Both ABF and HAADF were obtained with the illumination semi-angle of 25 mrad and probe current of 100 pA. The dwell time for imaging was set at 10 ms per pixel to ensure desirable signal to noise ratio. The collection angles for the ABF and HAADF images were fixed at 12–25 and 90–250 mrad, respectively. EDXS was performed to locate elemental distribution of Mo, P, and Pd with an SDD-type EDX detector. The attainable energy-resolution of the EDX detector is 130 eV. A higher beam current of 300 pA was used with a longer dwell time of 0.1 ms per pixel repeated for 200 times. Spatial drift was corrected with a simultaneous image collector. Mo L-edge, P K-edge and Pd L-edge were used for elemental mapping.

### Power X-ray diffraction

PXRD data were obtained on a PANAnalytical X’Pert Pro diffractometer in reflection mode at 40 kV and 40 mA using Cu Kα radiation.

### Raman spectroscopy

Raman spectra were recorded on a Renishaw Raman spectrometer at a laser excitation wavelength of 633 nm.

### X-ray absorption data collection and analysis

Mo K-edge and Pd K-edge X-ray absorption spectra were acquired under ambient condition at beamline BL14W1 of Shanghai Synchrotron Radiation Facility (SSRF) using a Si(311) double-crystal monochromator in transmission and fluorescence modes, respectively. The storage ring of SSRF was operated at 3.5 GeV with a maximum current of 250 mA. The energy was calibrated using Mo/Pd foil. While the incident and transmitted X-ray intensities were monitored by using standard ion chambers, the fluorescence signal was detected by using a 7 element Ge detector. To prevent air oxidation, the samples were prepared in a glove box, and they were uniformly mixed with BN powder and pressed to a pellet, which was sealed in a cell holder with Kapton windows for XAFS measurement. The XAFS raw data were background-subtracted, normalized, and Fourier transformed by the standard procedures with the ATHENA program^39, 40^. Least-squares curve-fitting analysis of the EXAFS *χ*(*k*) data was carried out using the ARTEMIS program^39^ with the theoretical scattering amplitudes, phase shifts, and the photoelectron mean-free path for all paths calculated by ab-initio code FEFF9.05^41^. The details of curve fitting are discussed in Supplementary Figs. 7–10 and Supplementary Tables 2 and 3. The Pd K-edge theoretical XANES calculations were carried out with the FDMNES code in the framework of real-space full multiple-scattering scheme using Muffin-tin approximation for the potential^41–43^. The energy-dependent exchange-correlation potential was calculated in the real Hedin-Lundqvist scheme, and then the spectra are convoluted using a Lorentzian function with an energy-dependent width to account for the broadening due both to the core-hole width and to the final state width. Satisfactory convergence for cluster sizes had been achieved.

### Density functional theory calculations

The electronic structure calculations were performed using the density functional theory and a plane-wave basis set as implemented in the VASP code^44^. The electron–ion interaction was treated by the projector-augmented wave method^45^ with the outmost *s*, *p*, and *d* states as valence orbital. The electron exchange-correlation energy was described by the modified Perdew-Burke-Ernzerhof generalized gradient approximation for solids (PBEsol)^46^. The kinetic energy cutoff was set to 350 eV, and the Brillouin zone was sampled in Monkhorst-Pack *k* point meshes with an interpolation grid spacing of 0.04 Å^–1^ for the 240 atoms supercell, to achieve the total energy convergence of less than 0.001 eV/atom. Structural relaxation was allowed until the force on each atom was <0.01 eV/Å.

### Catalysis testing in batch reactions

Phenol (18.4 mg, 0.195 mmol) was added into a stainless steel Parr autoclave (reactor volume, 50 mL) with decalin (7 mL), together with Pd/m–MoO_3_–P_2_O_5_/SiO_2_ (10.0 mg) and was stirred for 10 min. The autoclave was then sealed, purged with H_2_ for three times, and then placed under 1 MPa H_2_ at room temperature. The reaction was carried out at different temperature for a certain time with a stirring speed of 800 rpm. After reaction, the organic product was collected and analyzed by GC-MS. For the re-usability experiments using the Pd/m–MoO_3_–P_2_O_5_/SiO_2_ catalyst; after each reaction cycle, the spent catalyst was separated by centrifugation and used for the next run.

### Catalysis testing in fixed bed reactions

The fixed bed reaction was carried out in a HEL made continuous trickle bed reactor (mode FlowCAT) with both the liquid feed and hydrogen gas passing in downward direction. The Pd/m–MoO_3_–P_2_O_5_/SiO_2_ catalyst (127 mg) was located in the middle of the tubular reactor with quartz wool plugs on both the sides. Liquid feed was prepared by dissolving phenol in decalin to form a solution of 3.6 mg/mL. The reaction was carried out at 433–453 K (Fig. 3d and Supplementary Fig. 19), 1 Mpa with H_2_ flow rate of 10 cm^3^(STP)min^–1^ and liquid flow rate of 0.05–0.1 mL min^–1^ (Fig. 3d and Supplementary Fig. 16). The liquid was preheated at the desired reaction temperature before being fed into the reactor. The products were periodically collected from the outlet stream throughout the reaction and were analyzed by GC-MS. WHSV was calculated by dividing the feed flow rate per hour by weight of catalyst. The spent catalyst (32 h-test) was collected and protected in N_2_, and then quickly prepared as samples for further characterizations. For the re-usability of the Pd/m–MoO_3_–P_2_O_5_/SiO_2_ catalyst, the spent catalyst was not removed from the reactor but calcined on-site in air at 673 K for 5 h with a flowing rate of 20 cm^3^/min and then reduced in H_2_ at 323 K for 3 h with a flowing rate of 75 cm^3^/min.

### GC-MS analysis

GC-MS analysis was conducted by using an Agilent gas chromatograph equipped with an Agilent 19091N-133 column of mode HP-INNOWax with high polarity, 30 m × 250 μm × 0.25 μm connected online to mass spectrometer. The GC oven was programmed as: hold at initial temperature of 313 K for 5 min, ramp at 15 K min^–1^ to 523 K and hold at 523 K for 5 min. 2-isopropylphenol was used as the internal standard. The peaks were analyzed by comparing the corresponding spectra with those of the NIST 2011 MS library.

### WIBO analysis and conversion

The WIBO was derived from fast pyrolysis of grounded eucalyptus wood chip and bark. Wood log (wood chip + bark) was fed directly into pyrolysis reactor in forms of grounded wood and bark. Pyrolysis was carried out at 773 K in absence of oxygen. The produced bio-oil was separated into water-soluble phase and water-insoluble phase. For the WIBO part, the volatile species, mainly lignin-derived monomers, were determined by dissolving 84.0 mg WIBO and 4.03 mg internal standard, 2-isopropylphenol, in 1.5 mL methanol or only WIBO in decalin, and analyzing by GC-MS, as presented in Supplementary Fig. 29 and Fig. 5a. For the analysis of sugar component, 1.038 g WIBO was washed with 30 mL water while stirring at room temperature for 12 h, and the resulting water extraction was analyzed by HPLC, as presented in Supplementary Fig. 30. HPLC analysis was conducted by using a Laserchrom HPLC system equipped with a Aminex HPX 87H 300 × 7.8 column and Refractive Index Detector at 308 K using 0.025 M H_2_SO_4_ as the mobile phase with flow rate of 0.6 mL min^–1^. To hydrolyze oligomers into monomeric sugars, 200 μL concentrated H_2_SO_4_ was added into 2 mL upper water solution and heated at 393 K for 1 h. The resultant was also analyzed by HPLC, as presented in Supplementary Fig. 30.

For reaction at 453 K, WIBO (86.2 mg), decalin (7 mL), and Pd/m–MoO_3_–P_2_O_5_/SiO_2_ (170.8 mg) were added into a stainless steel Parr autoclave (reactor volume of 50 mL) and stirred for 10 min. Then, the autoclave was sealed, purged three times with H_2_, and placed under 1 MPa H_2_ at room temperature. The reaction was carried out at 453 K for 4 h with a stirring speed of 800 rpm. After reaction, the organic product was collected and analyzed by GC-MS using an added internal standard, as presented in Supplementary Fig. 32. For reaction at 523 K, 87.2 mg WIBO was used and the other parameters were kept identically. The reaction was carried out at 523 K for 15 h. The reaction products were analyzed by GC-MS, as presented in Supplementary Fig. 33.

The mass of lignin-derived phenols and furans in the WIBO and alkanes in the product were determined by internal standard. The corresponding response factors were determined by analyzing mixtures of those pure, commercial compounds of phenols, furans and alkanes, and internal standard with given weights. Mass yields of alkanes were calculated by the equation: mass of alkanes/mass of dried WIBO input. Carbon yields were calculated by the equation: mass of carbon in alkanes/mass of carbon in the WIBO input.

### Gel permeation chromatography

GPC measurements were performed on a Shimadzu LC-20AD instrument using MALLS detector. Two Mixed Bed PSS SDV linear S columns were used in series with tetrahydrofuran (THF) as mobile phase with flowing rate of 1.0 cm^3^ min^–1^ at 303 K. The MALLS detector was calibrated using a polystyrene standard. The sample was prepared by dissolving WIBO in THF with 1 wt% concentration and then filtering with 0.20 μm pore size microfilter.

### Thermogravimetric analysis (TGA)

TGA measurements were performed on a Mettler Toledo TGA/DSC 1 system. 16.76 mg of sample was heated in a corundum crucible between 423 and 973 K at a heating rate of 5 K/min in a flowing 50% O_2_/N_2_ mixture.

### TMPO-adsorbed sample preparation for ^31^P MAS NMR spectroscopy

100 mg of the catalyst was placed in a home-made glass tube, activated at 298 K for 2 h under vacuum (10^–1^ Pa), mixed with a CH_2_Cl_2_ solution containing 0.1 M TMPO under nitrogen, treated by ultrasound for 1 h (for equilibrium), and then the solvent was evacuated under vacuum. Finally, the sample tube was sealed for storage and transferred to Bruker 4 mm ZrO_2_ rotor with a Kel-F endcap in a glove box under nitrogen atmosphere before NMR spectroscopy measurement.

### ^31^P MAS solid-state NMR spectroscopy experiments

Solid-state MAS NMR spectroscopy experiments were carried out using a Bruker Avance III 400WB spectrometer at room temperature. To remove the effect of proton spins on quantitative ^31^P spectra (i.e., Fig. 4a), a strong radio frequency field (B) is usually applied at the resonance frequency of the non-observed abundant spins (^1^H herein), which contribute to the coupling of both spin species. The high power decoupling was thus used for the quantitative ^31^P analysis. Considering the long relaxation time of ^31^P nuclei in NMR spectroscopy experiment, 30° pulse with 1.20 μs width and 15 s delay time. The radiofrequency for decoupling was 59 kHz. The spectral width was 400 ppm, from 200 to −200 ppm. The number of scanning was 800 and spinning frequency was 10 kHz. The ^31^P chemical shifts were reported relative to 85% aqueous solution of H_3_PO_4_, with NH_4_H_2_PO_4_ as a secondary standard (0.81 ppm). The quantitative analysis of adsorbed TMPO molecules was then calculated according to the calibration line established by running standard samples with various adsorbed TMPO concentration^47, 48^. On the other hand, for samples without adsorbing TMPO (Supplementary Fig. 4), the ^1^H*-* ^31^P cross-polarization was used.

### Surface area measurements

Specific surface areas were analyzed by Micromeritics Tristar II. The samples were measured for the N_2_ adsorption and desorption at 77 K. Before each measurement, the samples were first in-situ degassed overnight at 110 °C for 12 h.

### Electron paramagnetic resonance

Continuous-wave electron paramagnetic resonance (CW-EPR) was carried out in the Centre for Advanced Electron Spin Resonance (CAESR), on two spectrometers and frequencies. X-band measurements (ca. 9.4 GHz) were performed on a Bruker BioSpin EMXmicro spectrometer with a Bruker SHQE-W cylindrical TE_011_-mode resonator. The temperature was controlled with an Oxford Instruments ITC-503 cryostat and ESR-900 cryostat. W-band measurements (ca. 94 GHz) were performed on a Bruker BioSpin EleXsys E680 spectrometer with a Bruker EN600-1021H cylindrical TE_011_-mode resonator. The temperature was controlled with an Oxford Instruments ITC-503 cryostat and CF-935O cryostat. For W-band, the microwave frequency was 93.9373 GHz for W-band, microwave power 40 μW, modulation amplitude 10 G, with a sweep of 84 s over 450 mT. For X-band, the microwave frequency was 9.38914 GHz, microwave power 4 mW, modulation amplitude 6 G, with a sweep of 41 s over 80 mT. The field modulation frequency was 100 kHz at both microwave frequencies. Data analysis was performed in Matlab, and EPR simulations employed the EasySpin toolbox^49^.

In temperature-dependent X-band EPR measurements the sample does not undergo microwave power saturation down to 20 K, while measurements present here were acquired at 85 K. Quantitative X-band CW-EPR measurements determined a total spin concentration of 9.1 × 10^16^ spins/mg, and double-integration of best-fit W-band simulations shows that the Mo^5+^ accounts for 80% of EPR-active species. W-band simulations employed a g-strain linewidth model of *σ*_⊥_ = 0.0054 and *σ*_||_ = 0.0079, which the same as at X-band. Simulations uncertainties are estimated in g-values as +/− 0.001 and hyperfine as +/− 5 MHz. Hyperfine values reported as *A*(^95^Mo) are those of ^95^Mo (15.9% natural abundance), while the ^97^Mo isotope (9.56% natural abundance) is related to ^95^Mo by the ratio of nuclear g-factors (−0.3734(^97^Mo)/−0.3657(^95^Mo)).

For EPR results, at 94 GHz, W-band (Supplementary Fig. 22), a signal consistent with six-coordinate Mo^5+^ is resolved from oxygen vacancies at *g* = 2 and donor defects at *g* = 1.977 with an axial g-matrix of *g*_⊥_ = 1.933 and *g*_||_ = 1.884^50^. Natural abundance ^95/97^Mo^5+^ hyperfine in X-band (Supplementary Fig. 23), 9.4 GHz, point to at least two species in the precursor, which are similar to two of three components in the H_2_-reduced form, but with about one-fourth overall EPR intensity in the precursor. A third component of reduced catalyst arises as an additional *g*_||_ feature at *g*~1.90 and an additional component of hyperfine in the high field *A*_||_(^95/97^Mo) feature, as demarcated in Supplementary Fig. 24. The W-band sample, aged under air, results in a relative reduction of the *g* = 1.90 feature, indicating that the corresponding site is likely surface-exposed. Multi-component simulations in Supplementary Fig. 24 also show an increase in *g*_||_ from the precursor value by Δ*g* of ca. 0.0025 upon reduction, on the trend of increasing ionic ligand character.

### Superconducting quantum interference device (SQUID) magnetometry

Magnetic susceptibility measurements of powdered samples of catalyst before and after H_2_ reduction were carried out using a Quantum Design MPMS-5 SQUID magnetometer at a field of 0.1 T and different temperatures (2–300 K). Accurately weighed samples (ca. 60 mg) were placed into gelatine capsules and then loaded into nonmagnetic plastic straws before being lowered into the cryostat. No corrections were made for the diamagnetic contribution to the magnetic moment due to the excess silica, and it was assumed the paramagnetic contribution originated solely from Mo^5+^ present in the sample, as determined by ICP-MS. Hence, a total magnetic susceptibility per Mo is reported. For quick comparison the magnetic susceptibility (per Mo) at 2 K, for catalyst before and after reduction is 6.73 × 10^–4^ and 1.19 × 10^–3^ cm^3^ mol^−1^, respectively, demonstrating the formation of more Mo^5+^ in the catalyst after H_2_ reduction.

### Temperature programmed reduction

TPR was performed on Micromeritics AutoChem II 2920 using a flow of H_2_ in N_2_ (10%, 50 mL min^–1^) ramping from 309 to 1073 K at a rate of 5.0 °K min^–1^.

### Diffuse-reflectance infrared Fourier transform spectroscopy

DRIFT spectroscopy studies were performed using a Bruker Tensor 27 spectrometer fitted with a high-sensitivity MCT detector (with resolution of 4 cm^−1^) and a diffuse IR heated chamber equipped with ZnS window. The reduced catalyst (Pd/m–MoO_3_–P_2_O_5_/SiO_2_) and reduced Pd/SiO_2_ with mass of 20 mg were pre-treated in the presence of 5% H_2_ in Ar at 383 K for 1 h. The oxidized catalyst and oxidized Pd/SiO_2_ (PdO/SiO_2_) with a mass of 20 mg were pre-treated in air at 573 K for 1 h and then cooled down to 383 K. The ZnS window was kept being heated to avoid phenol condensation. After the pre-treatments, the background was scanned in the presence of He at 383 K and then the spectra were collected in the presence of phenol/helium mixture at 383 K. The background and spectra were recorded at a resolution of 4 cm^−1^ with 128 accumulation scans.

### Data availability

Data that support the findings of this study are available within the article (and its Supplementary Information files) and from the corresponding authors on reasonable request.

## Electronic supplementary material

Supplementary Information
