# Supplementary material for: Hydrodeoxygenation of water-insoluble bio-oil to alkanes using a highly dispersed Pd–Mo catalyst
Source: Nat Commun. 2017 Sep 19;8:591. doi: 10.1038/s41467-017-00596-3 (PMC5605710; doi:10.1038/s41467-017-00596-3)
Supplement: Supplementary file 1 — Supplementary Information [file 41467_2017_596_MOESM1_ESM.pdf]

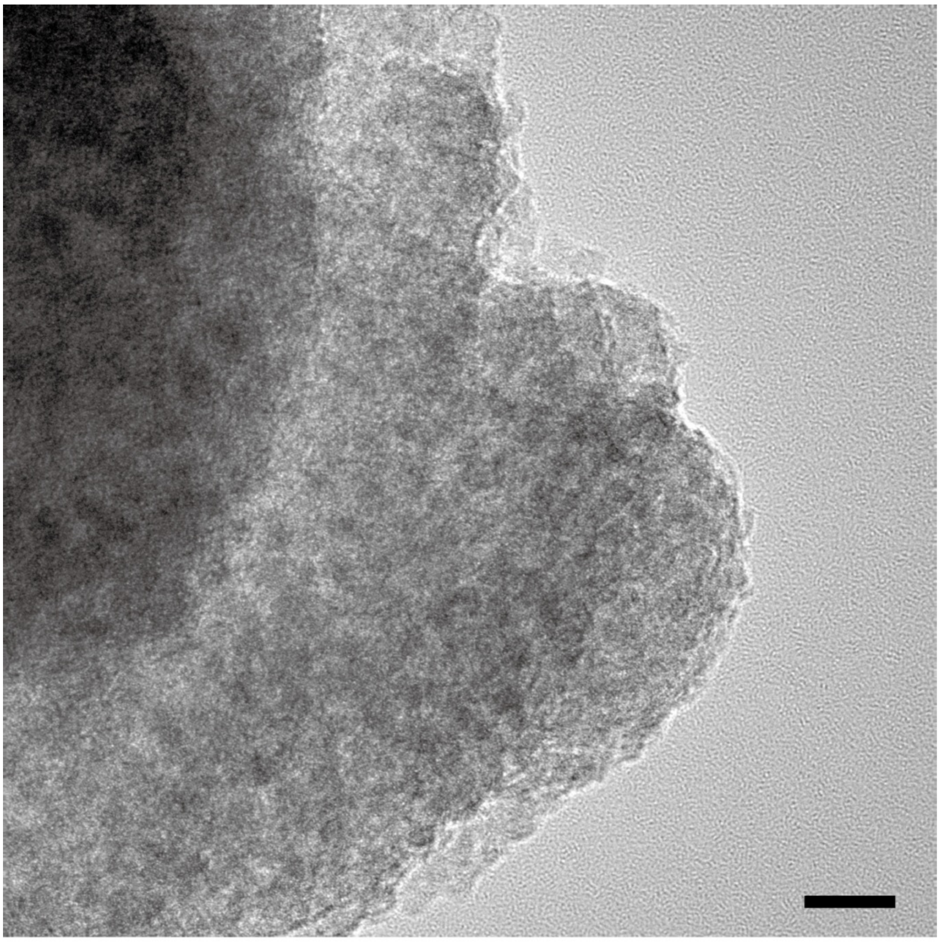

Supplementary Figure 1. Transmission electron microscopy of Pd/m-MoO_3-P_2O_5/SiO_2. Scale bar equals 10 nm.

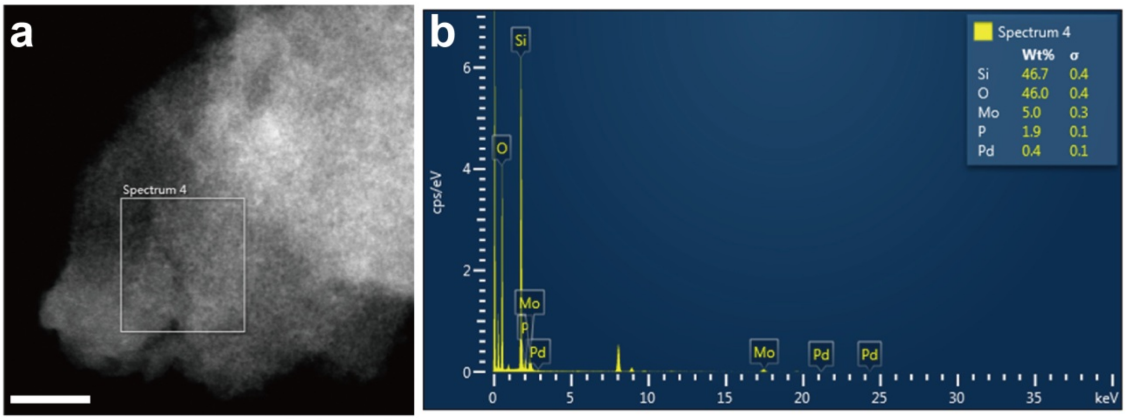

Supplementary Figure 2. a, Energy dispersive X-ray spectroscopy of a representative region of the Pd/m-MoO_3-P_2O_5/SiO_2 and **b**, corresponding HAADF-STEM image. According the inlet table, the molar ratio of Pd/Mo/P = 0.072/1.0/1.1. Scale bar equals 200 nm.

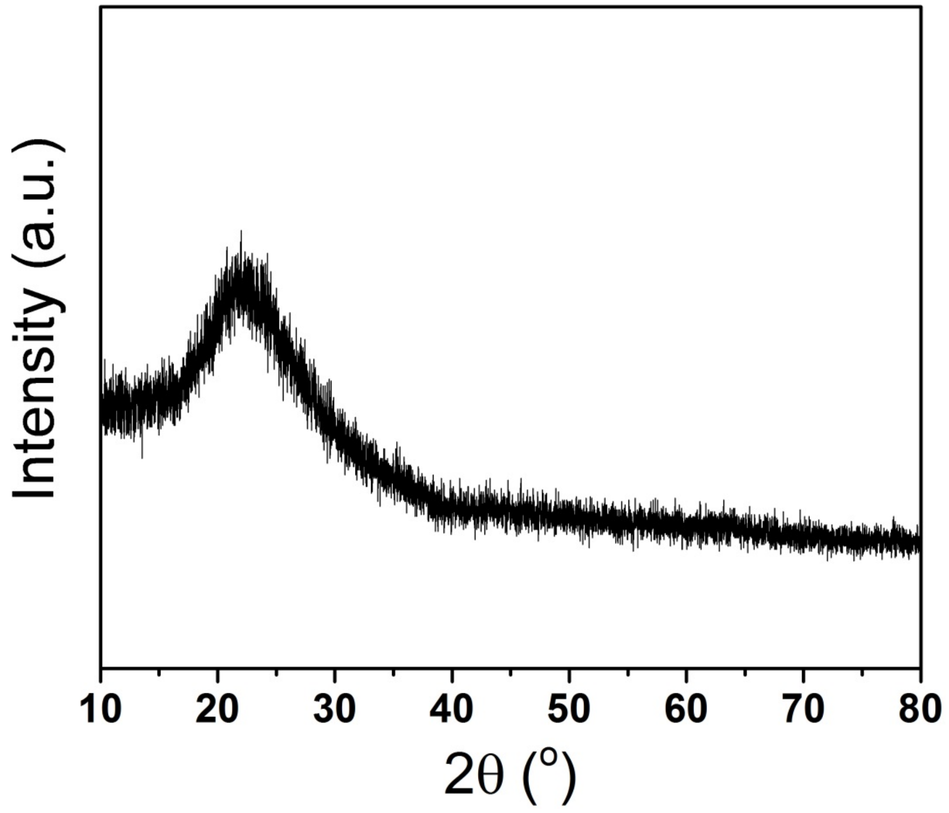

Supplementary Figure 3. X-ray diffraction pattern of the Pd/m-MoO_3-P_2O_5/SiO_2.

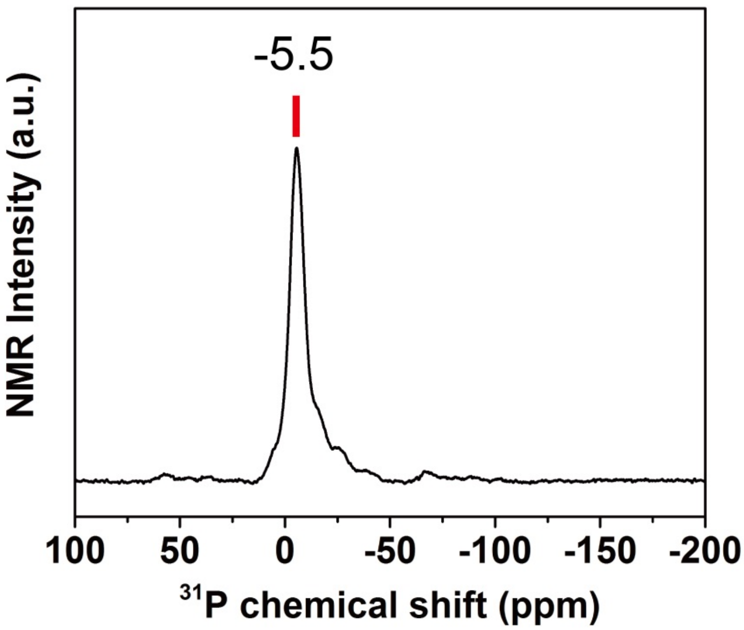

Supplementary Figure 4. Solid state ^{31}P MAS NMR spectra of Pd/m-MoO_3-P_2O_5/SiO_2.

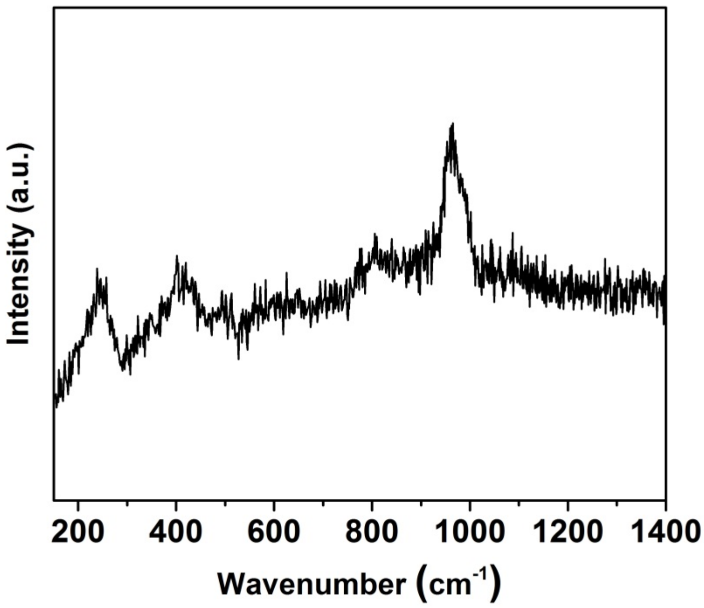

Supplementary Figure 5. Raman spectrum of Pd/m-MoO_3-P_2O_5/SiO_2.

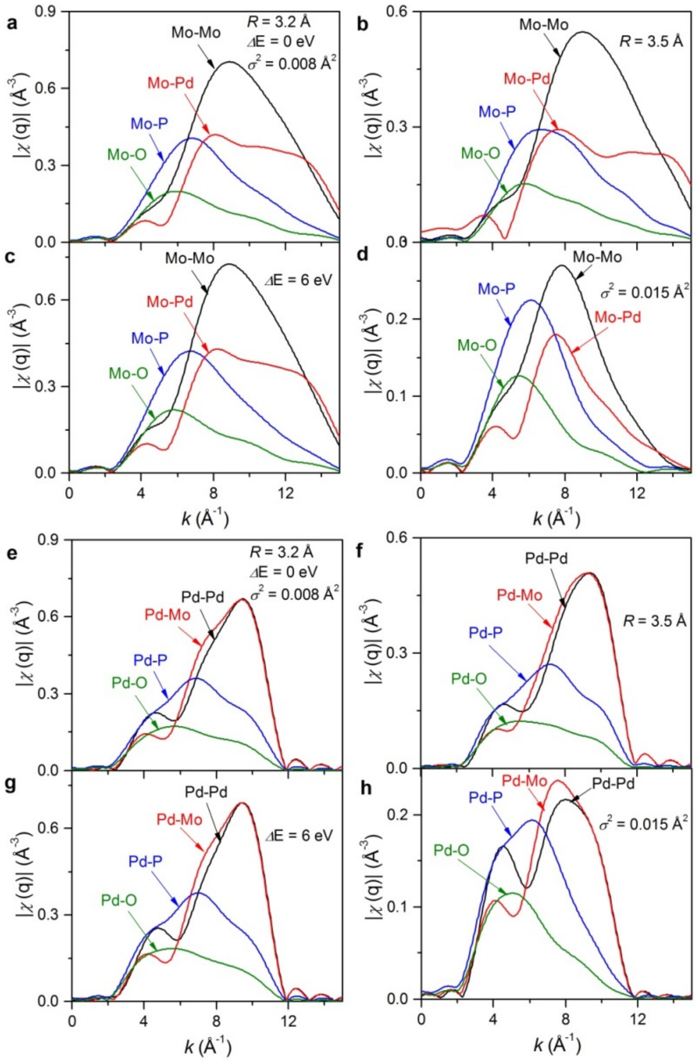

Supplementary Figure 6. Comparison of the q-space magnitudes for FEFF-calculated, k^3-weighted EXAFS paths. The wavelet transform maximum corresponds to the same location of the maximum in the q-space magnitude and is known to be affected by atomic number Z, the path length R, energy shift ΔE, and Debye–Waller factors σ^2, thus allowing qualitative interpretation of the scattering path origins for the wavelet transform maxima. **a**,**e**, The effect of atomic number Z (R, σ^2 and ΔE are fixed) on the paths. **b**,**f**, The effect of the path length R on the paths (σ^2 and ΔE are fixed). **c**,**g**, The effect of the energy shift ΔE on the paths (R and σ^2 are fixed). **d**,**h**, The effect of the Debye–Waller factors σ^2 on the paths (R and ΔE are fixed). It can be observed that the Mo-Pd/Mo path shows one maximum near 8.5 Å^{-1}, while Pd-Pd/Mo path shows one maximum near 9.5 Å^{-1} associated with a shoulder around 4.5 Å^{-1}. Besides, the Pd(Mo)-P/Si path shows one maximum near 7.0 Å^{-1} and the Pd(Mo)-O path shows one maximum near 5.5 Å^{-1}.

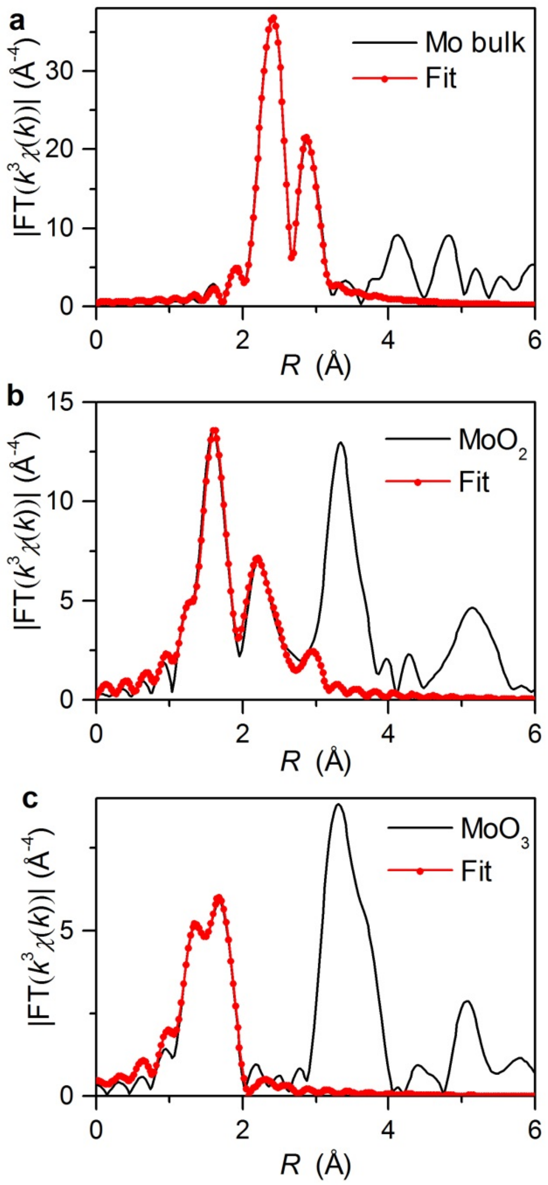

Supplementary Figure 7. Fourier-transformed magnitude of Mo K-edge EXAFS spectra in *R* space for **a**, Mo bulk, **b**, MoO_2, and **c**, MoO_3. Measured and calculated spectra are matched very well for all samples. The best-fit parameters are shown in Supplementary Table 2.

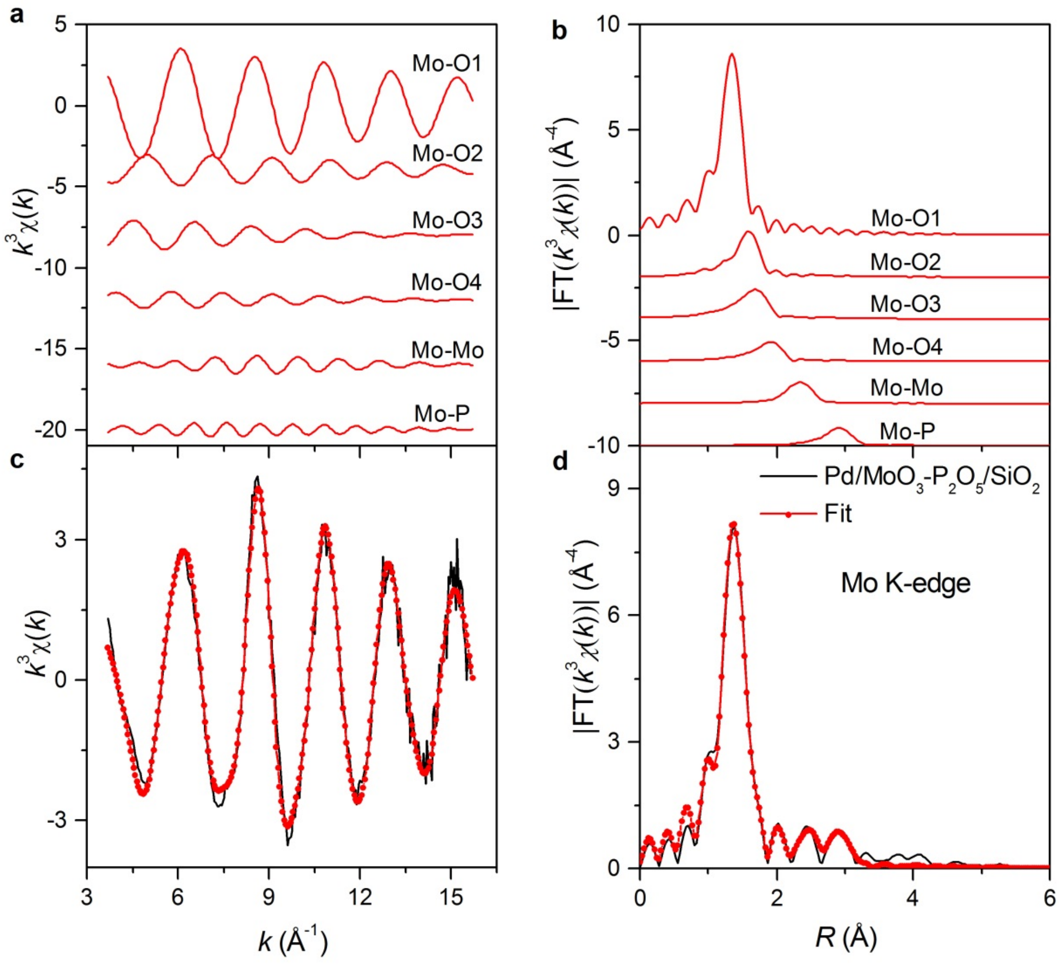

Supplementary Figure 8. Mo K-edge EXAFS analysis of Pd/MoO_3-P_2O_5/SiO_2 in *k* and *R* spaces, respectively. **a**,**b**, Mo-O1, Mo-O2, Mo-O3, Mo-O4, Mo-Mo, and Mo-P two-body back-scattering signals (red lines, curves from top to bottom). **c**,**d**, Total theoretical signal (dotted red line) superimposed on the experimental one (black line). Measured and calculated spectra are matched very well, and the best-fit parameters are shown in Supplementary Table 2.

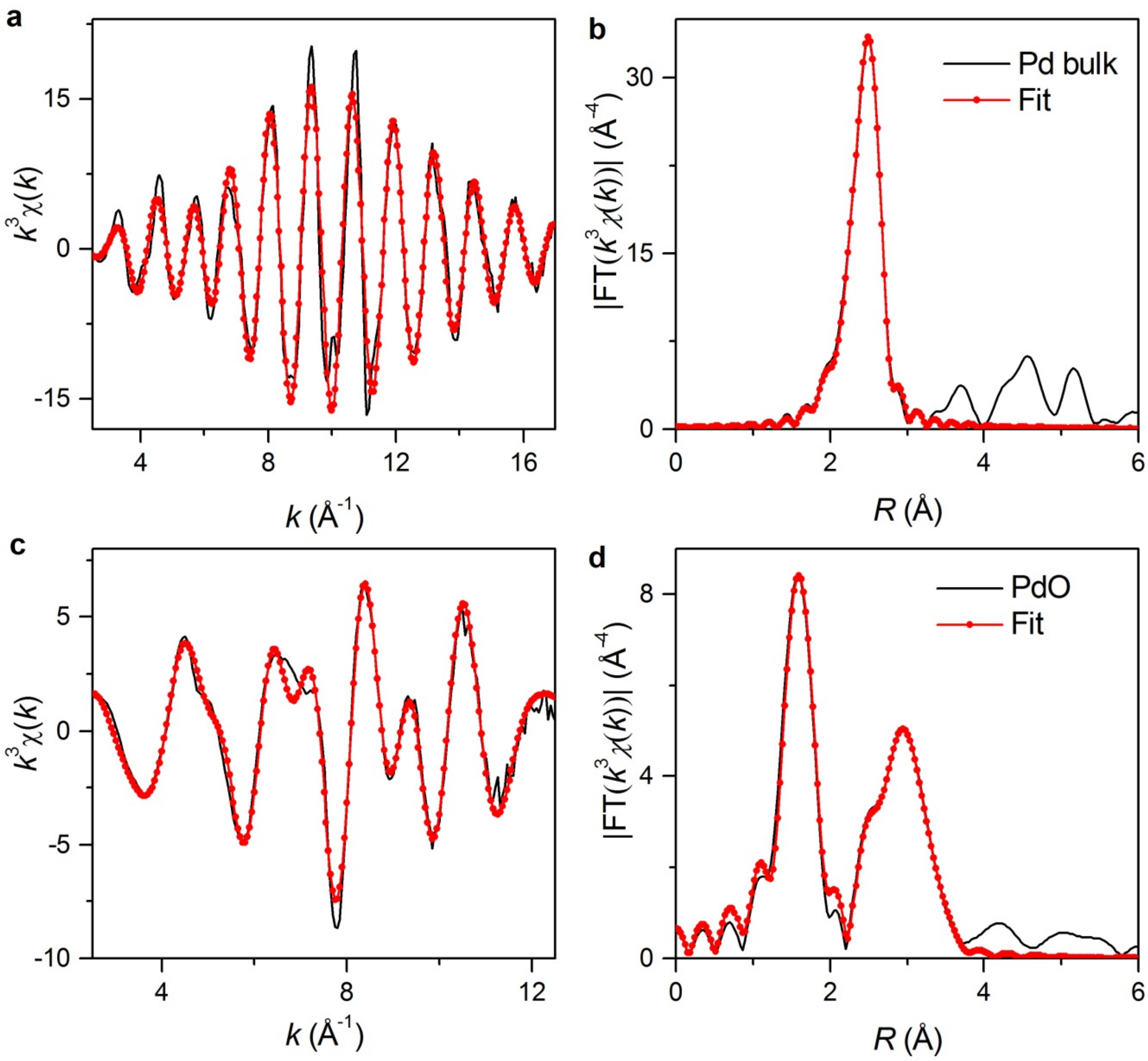

Supplementary Figure 9. k^3-weighted Pd K-edge EXAFS spectra and the Fourier-transformed magnitudes for **a**,**b**, Pd bulk and **c**,**d**, PdO. Measured and calculated spectra are matched very well for all samples. The best-fit parameters are shown in Supplementary Table 3.

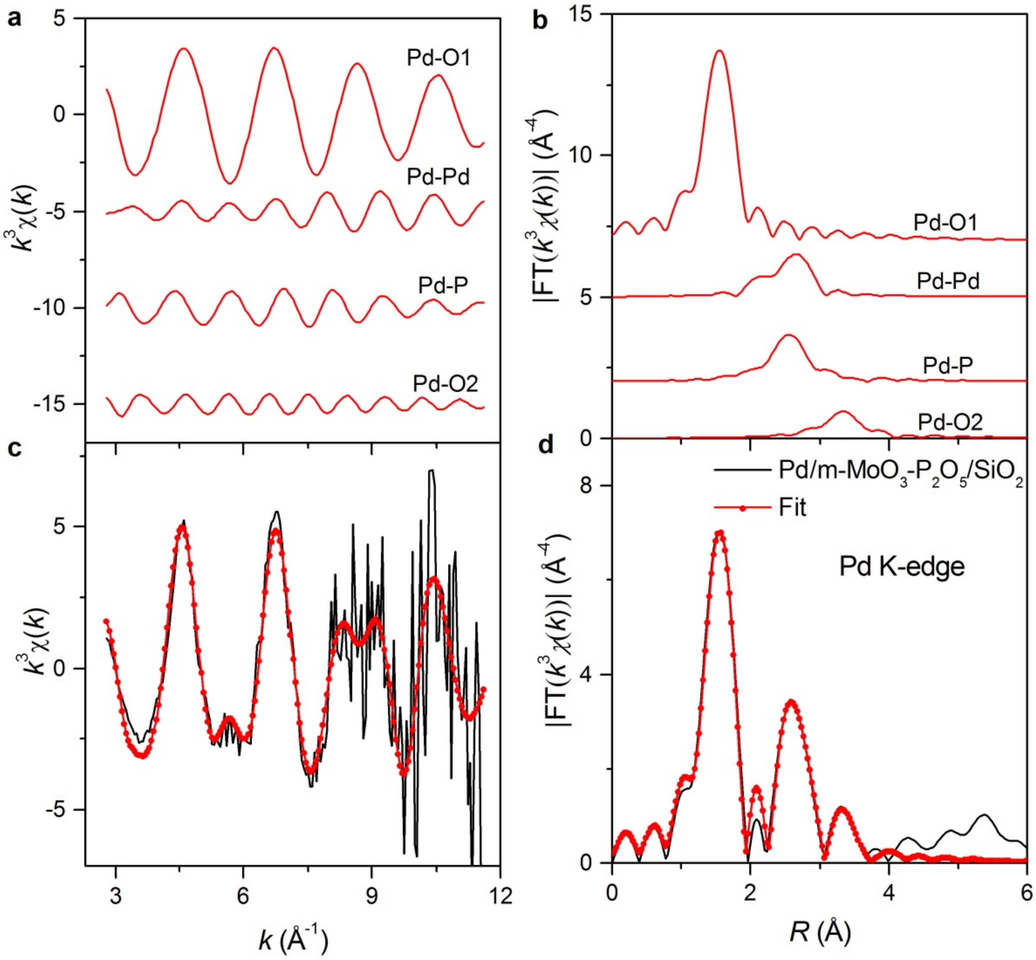

Supplementary Figure 10. Pd K-edge EXAFS analysis of Pd/m-MoO_3-P_2O_5/SiO_2 in *k* and *R* spaces, respectively. **a**,**b**, Pd-O1, Pd-Pd, Pd-P, and Pd-O2 two-body back-scattering signals (red lines, curves from top to bottom). **c**,**d**, Total theoretical signal (dotted red line) superimposed on the experimental one (black line). Measured and calculated spectra are matched very well, and the best-fit parameters are shown in Supplementary Table 3.

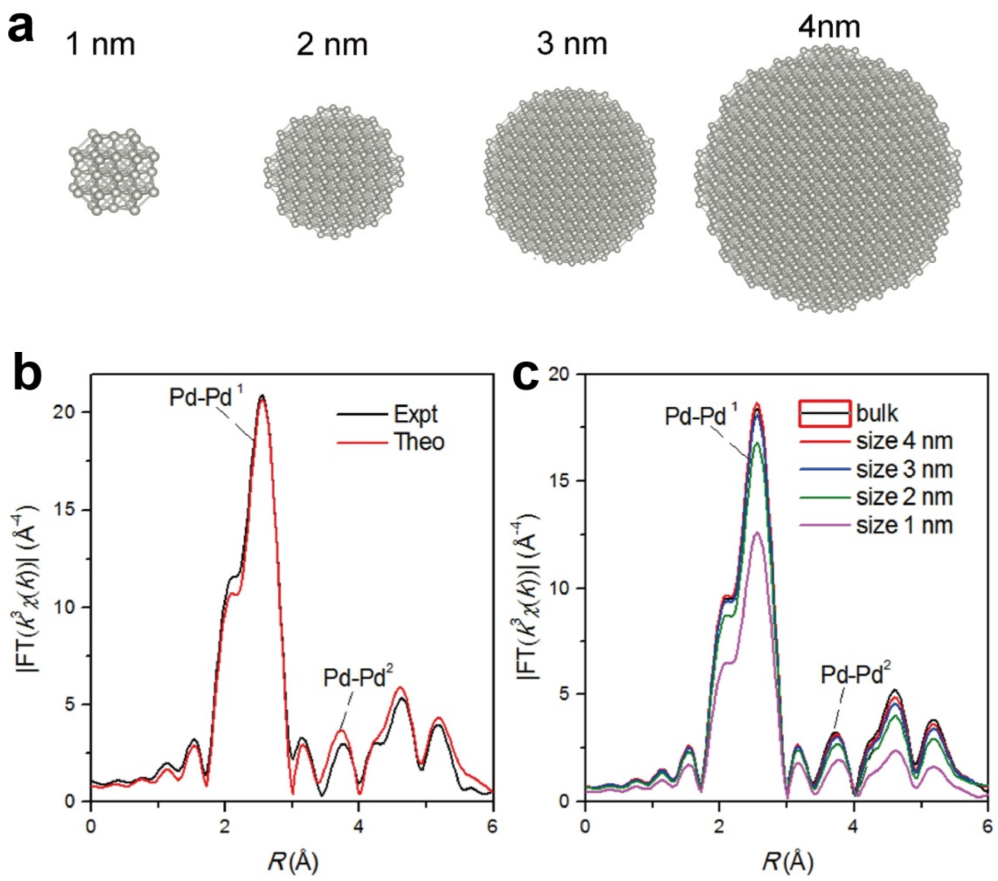

Supplementary Figure 11. a, Pd cluster models of different sizes. Comparison of Pd K-edge EXAFS results simulated for **b**, bulk Pd and **c**, Pd nanoclusters of different sizes. The first and second Pd-Pd coordination shells were denoted as $Pd\text{-}Pd^1$ and $Pd\text{-}Pd^2$, respectively. The EXAFS simulation was carried out with FEFF9.0 software, and the temperature effect is accounted for by using the correlated Debye model (the DEBYE card in FEFF9.0 is set with temperature of 300 K and Debye-temperature of 274 K). The total EXAFS result for a given Pd nanocluster is obtained via the sum of the contributions from all Pd atoms in the cluster.

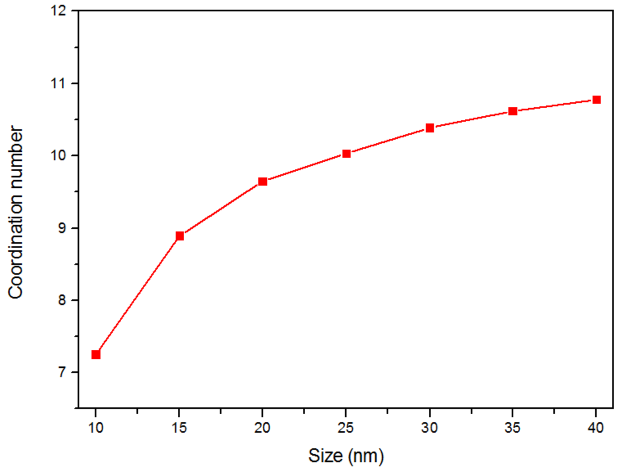

Supplementary Figure 12. The average Pd-Pd coordination number as a function of the Pd nancluster sizes. The Pd nanocluster is approximated as spherical in shape.

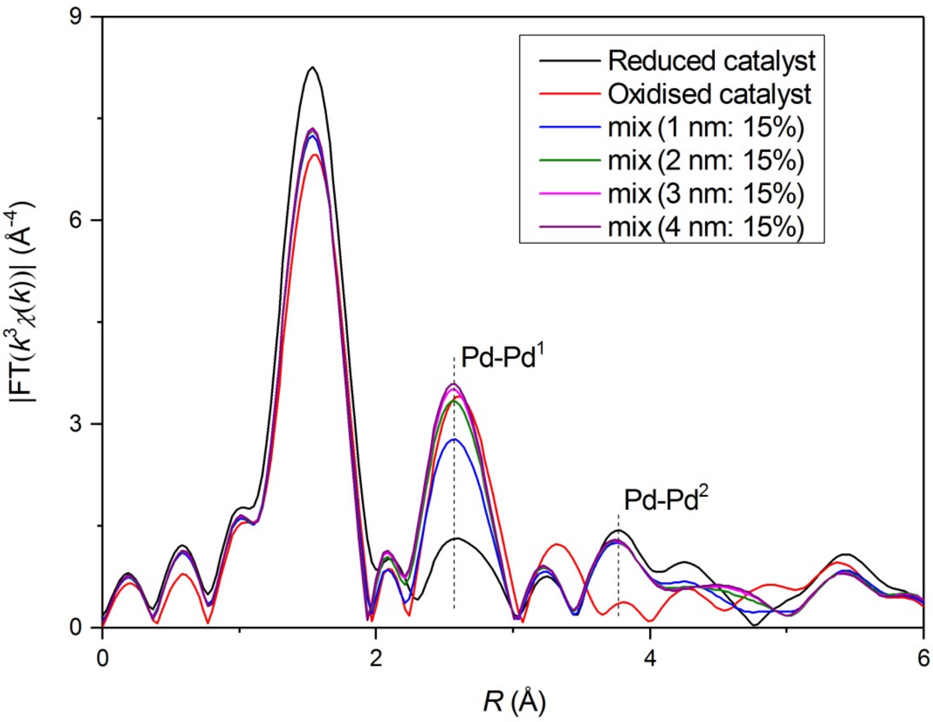

Supplementary Figure 13. Comparision between the experimental Pd K-edge EXAFS results of reduced catalyst and oxidised catalyst, as well as the weighted ones by mixing the experimental EXAFS signal for oxidised catalyst with the theoretical ones of Pd clusters by a weight ratio of 85%:15%. The first and second Pd-Pd coordination shells were denoted as Pd-Pd[1] and Pd-Pd[2], respectively.

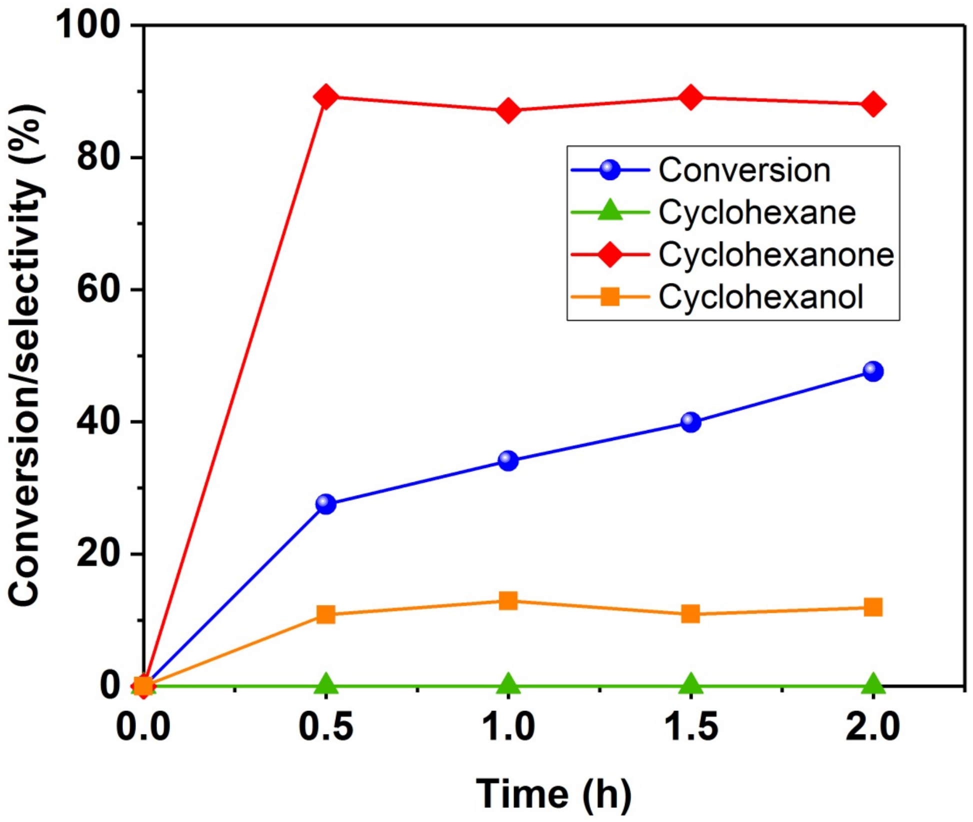

Supplementary Figure 14. Time dependence of the phenol conversion and product selectivity on the Pd/SiO_2 in a batch reaction. Reaction conditions: phenol (0.195 mmol), catalyst (including 0.00045 mmol Pd), decalin (7 mL), 383 K, 1 MPa H_2; reaction mixture stirred at 800 rpm.

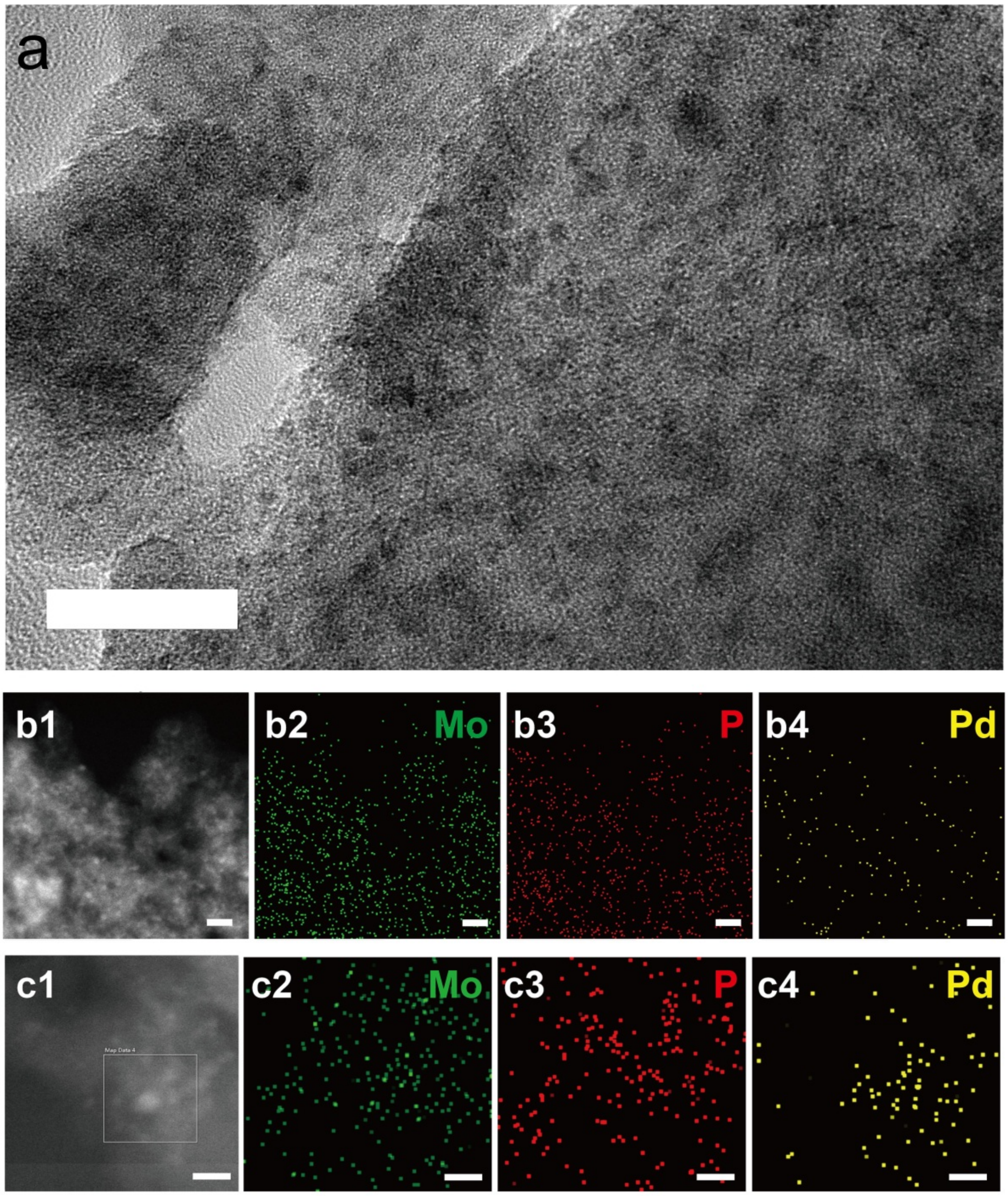

Supplementary Figure 15. **a**, TEM image, **b1-b4**, low-magnification and **c1-c4**, high-magnification STEM-EDS elemental mapping results of the spent Pd/m-MoO_3-P_2O_5/SiO_2 catalyst after fixed-bed reaction over 32 hours. Reaction conditions: 433 K, 1 MPa H_2 with flow rate of 10 cm^3(STP)min^{-1}, WHSV = 0.18 h^{-1}. **a**, scale bar equals 20 nm, **b1-b4**, scale bars equal 10 nm. **c1-c4**, scale bars equal 5 nm. The results suggest that there is no obvious change after reaction.

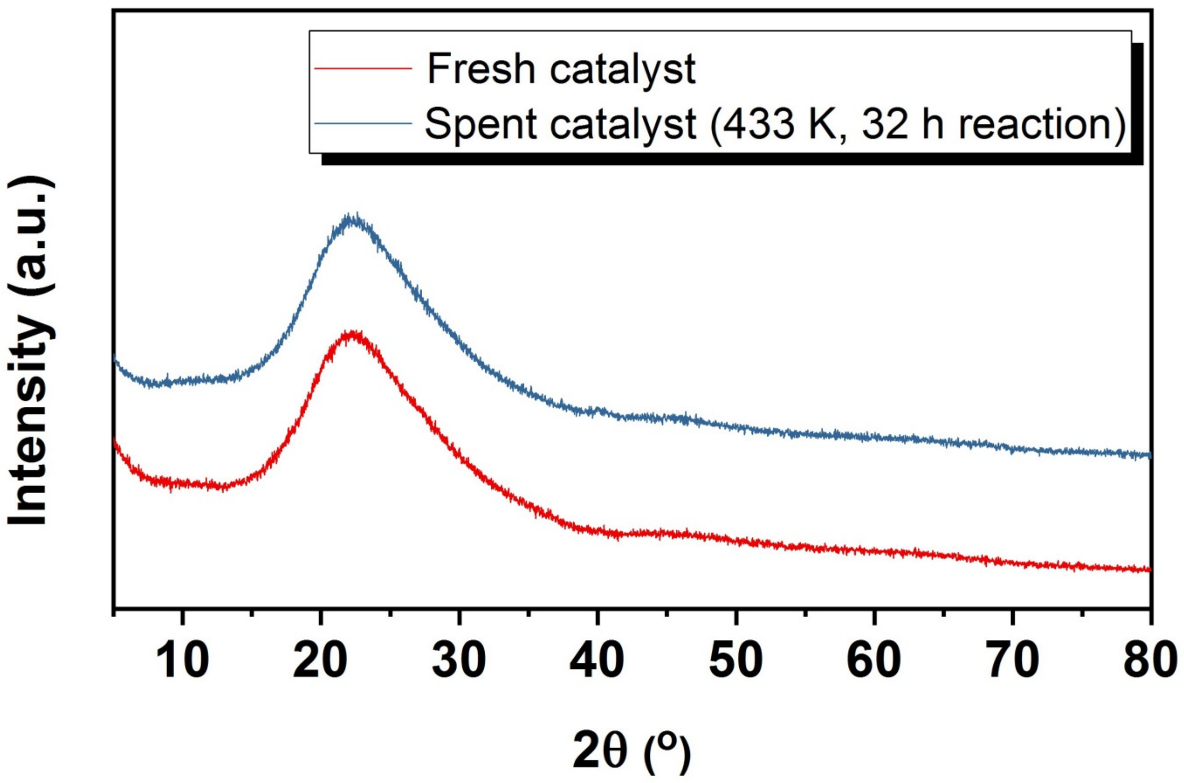

Supplementary Figure 16. X-ray diffraction pattern of the fresh Pd/m-MoO_3-P_2O_5/SiO_2 catalyst and spent catalyst after fixed-bed reaction over 32 hours. Reaction conditions: 433 K, 1 MPa H_2 with flow rate of 10 cm^3(STP)min^{-1}, WHSV = 0.18 h^{-1}. The lack of crystalline peak for Pd indicates that there is no obvious Pd aggregation or size growing up after reaction.

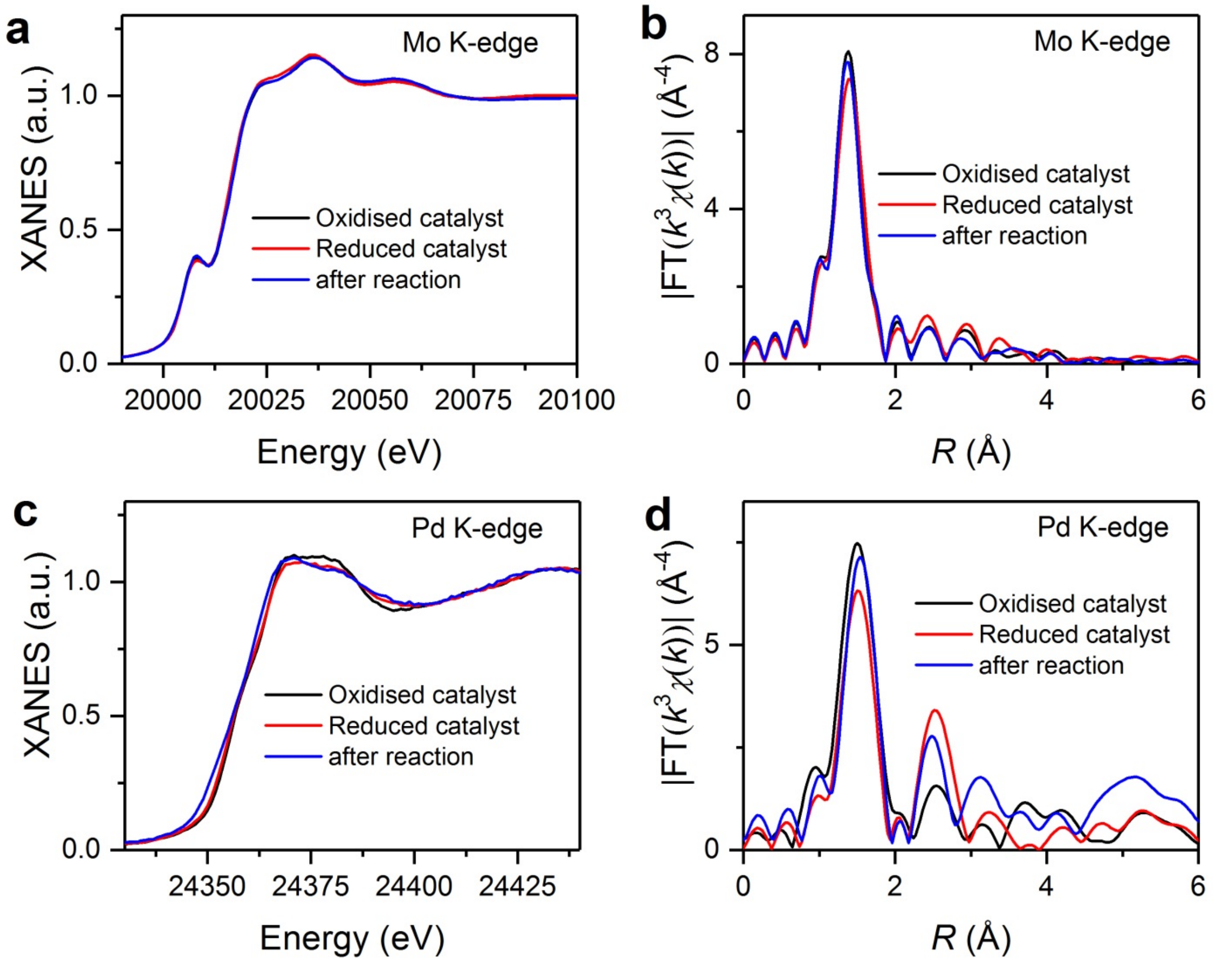

Supplementary Figure 17. Comparison of the experimental XANES spectra for **a**, Mo and **c**, Pd atoms, and EXAFS spectra for **b**, Mo and **d**, Pd atoms in the reduced catalyst, oxidised catalyst and that after fixed-bed reaction over 32 hours.

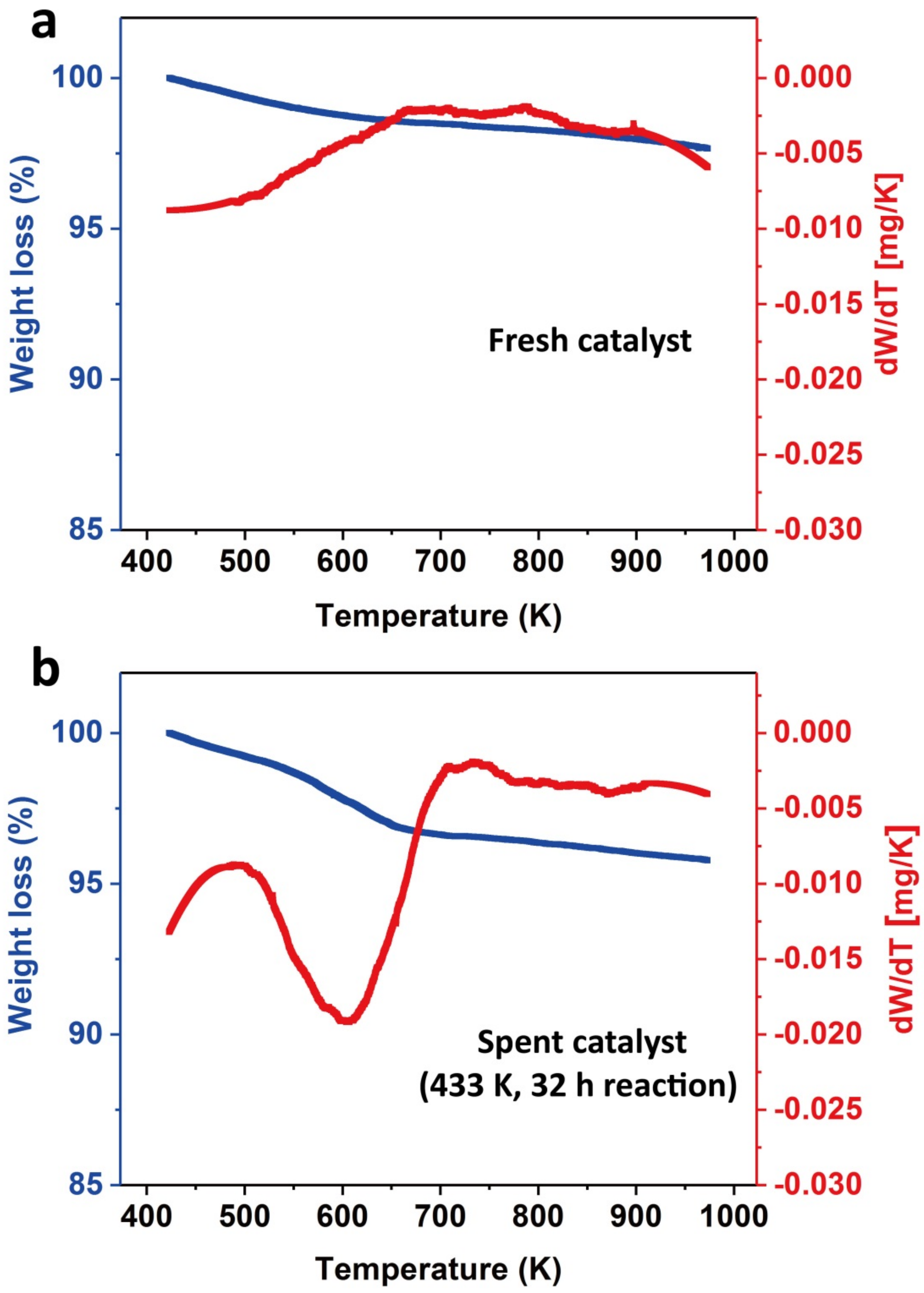

Supplementary Figure 18. Thermogravimetric analysis (TGA) of **a**, fresh catalyst and **b**, spent catalyst after fixed-bed reaction over 32 hours. Reaction conditions: 433 K, 1 MPa H_2 with flow rate of 10 cm^3(STP)min^{-1}, WHSV = 0.18 h^{-1}. The weight change during oxidation together with DTG profiles are shown in the figure. The profile of spent catalyst exhibits one intense peak at temperature between 503 and 703 K, which is attributed to the burning of carbonaceous residues.

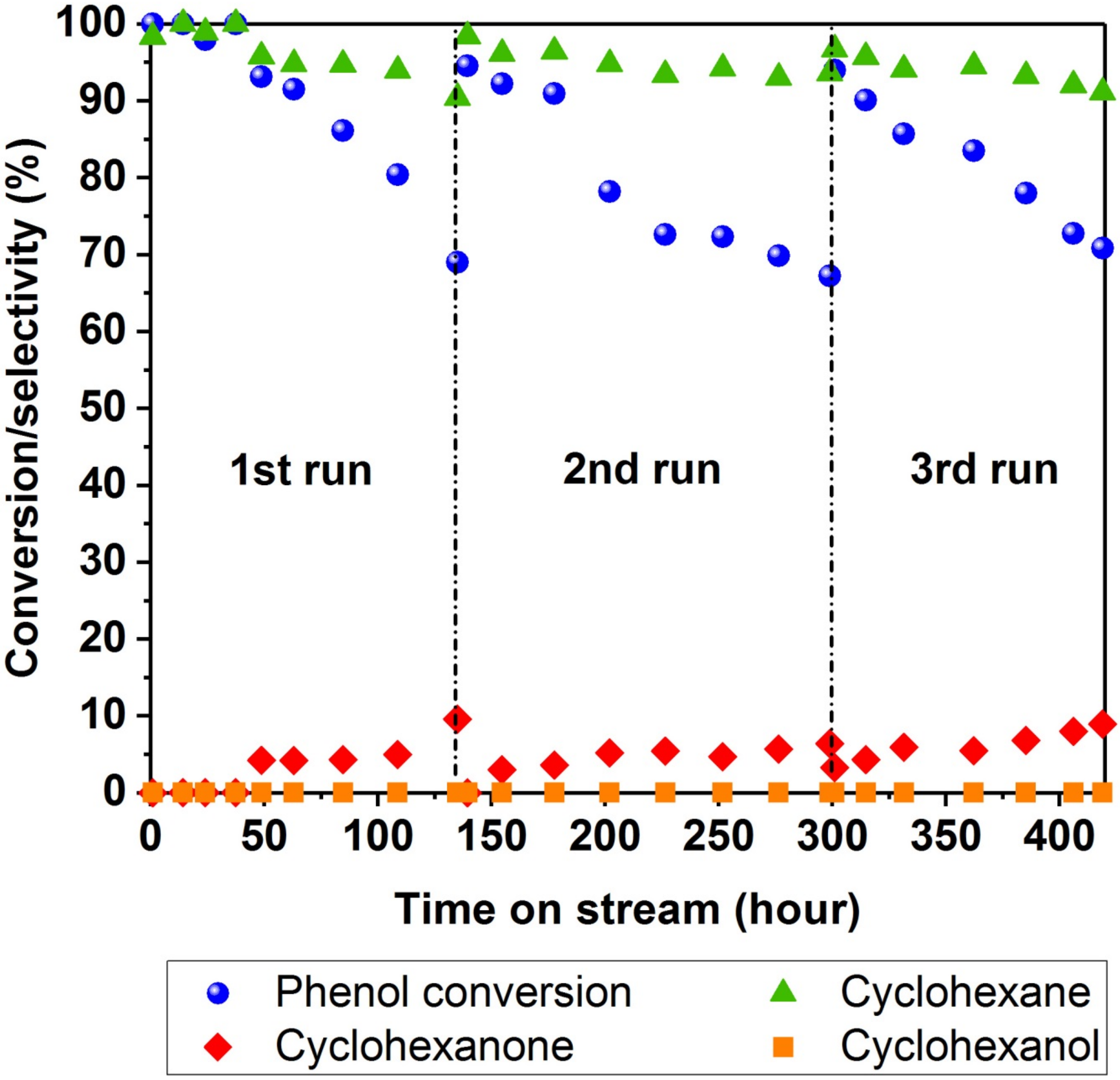

Supplementary Figure 19. Long-term stability and regenerability test of Pd/m-MoO_3-P_2O_5/SiO_2 in continuous flow reaction at 453 K, 1 MPa H_2 with WHSV of 0.085 h^{-1} for 3 cycles over 419 h. 1st run for 135 h, 2nd run for 164 h and 3rd run for 120 h. For the regeneration process, the spent catalyst was calcined in air flow at 673 K for 5 h and then reduced in H_2 flow at 323 K for 3 h.

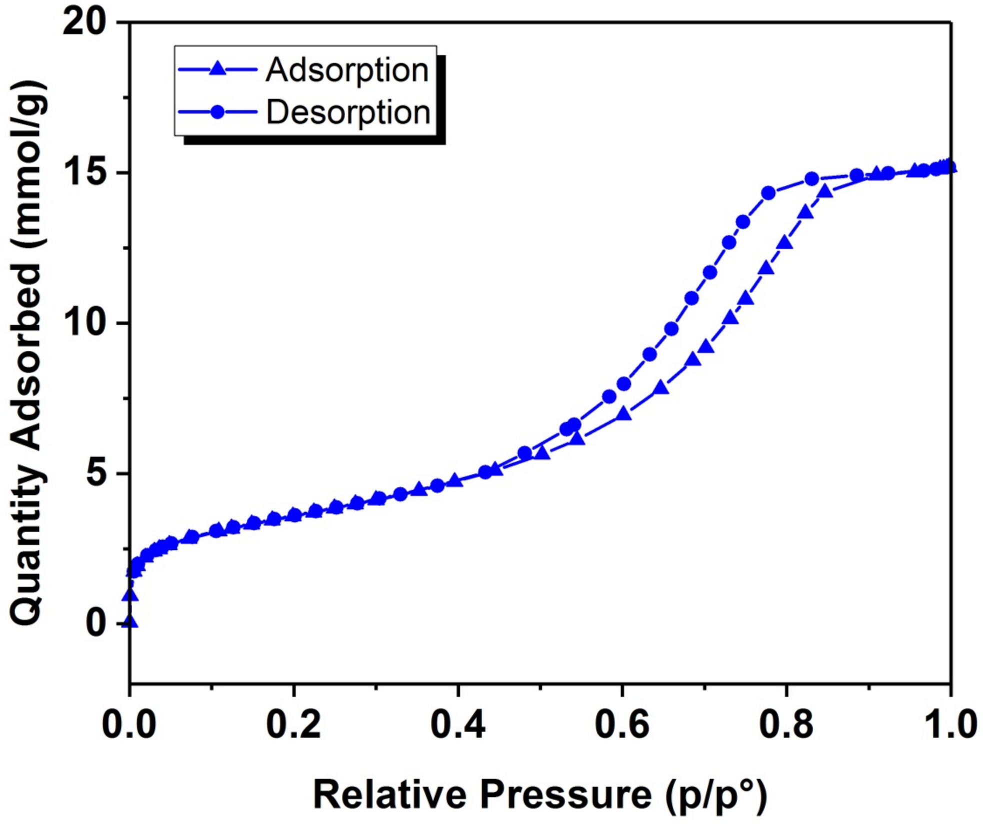

Supplementary Figure 20. N_2 adsorption/desorption isotherm of Pd/m-MoO_3-P_2O_5/SiO_2. The BET surface area is 291.5±0.4 m^2/g.

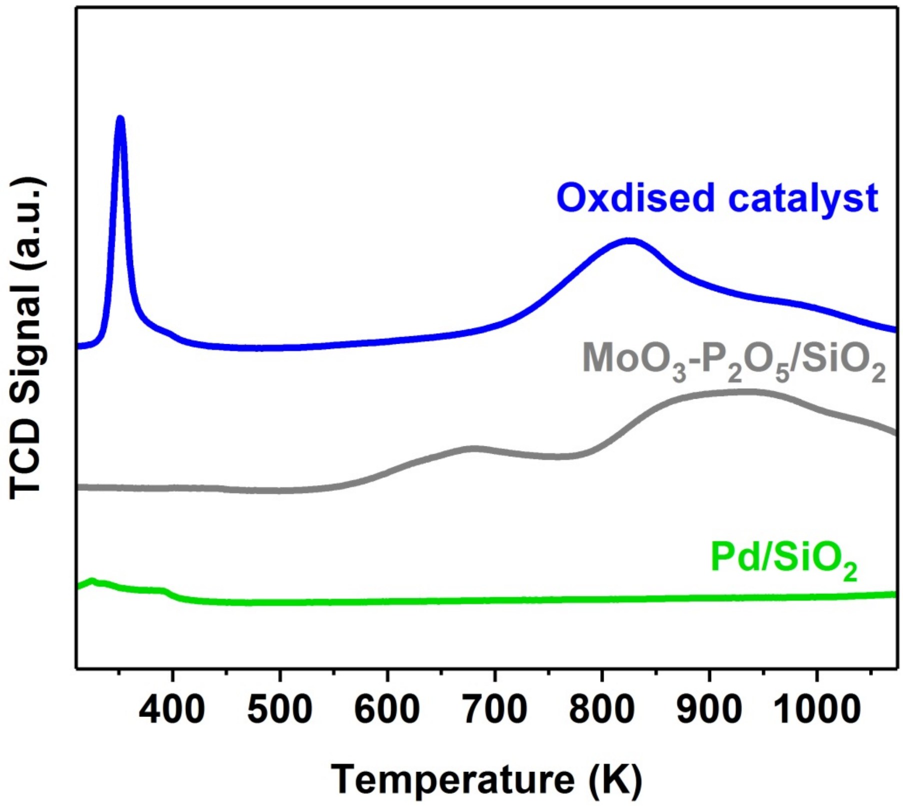

Supplementary Figure 21. TPR profile of the oxidised catalyst and comparing samples. It shows that only over the oxidised catalyst, an intense peak appeared at low temperature (ca. 351 K) which can be assigned to oxygen vacancy formation. This process was promoted by Pd facilitated H_2 dissociation and hydrogen atom spillover.[1]

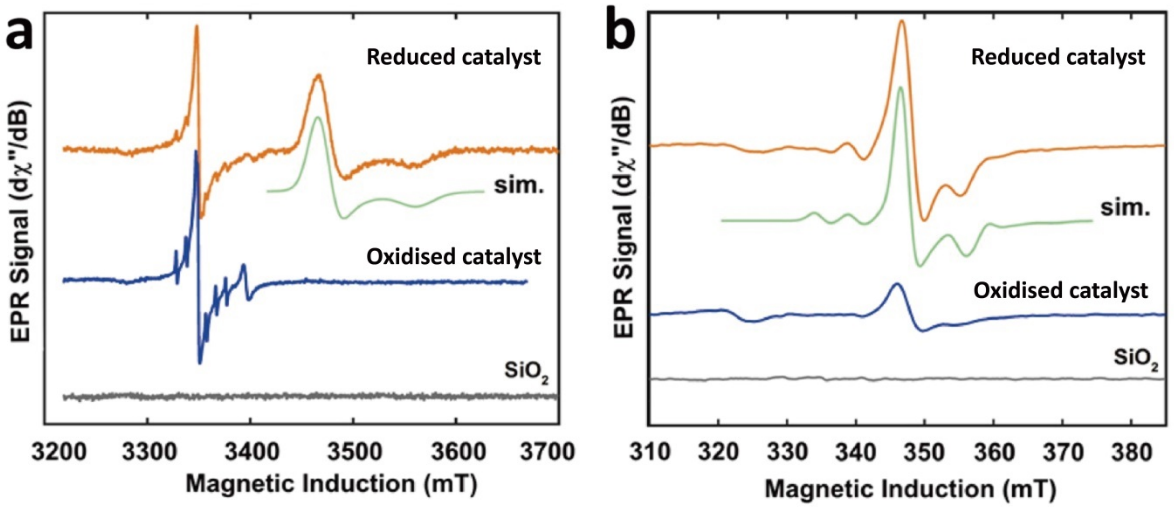

Supplementary Figure 22. CW-EPR at W-band in Panel **a**, and at X-band, in Panel **b**, of the oxidised catalyst and reduced catalyst (blue and orange, respectively), and the SiO_2 substrate (in gray). A simulation in the limit of a single Mo(V) species is shown in green with the g-values of $g_{\perp}$=1.9328 and g||= 1.8835 from W-band and an isotropic $A(^{95}Mo)$ hyperfine of 133 MHz determined by simulations of the X-band data. For W-band, the microwave frequency was 93.9373 GHz for W-band , microwave power 40 μW, modulation amplitude 10 G, with a sweep of 84 sec. over 450 mT. For X-band, the microwave frequency was 9.38914 GHz, microwave power 4 mW, modulation amplitude 6 G, with a sweep of 41 sec. over 80 mT. The field modulation frequency was 100 kHz at both microwave frequencies.

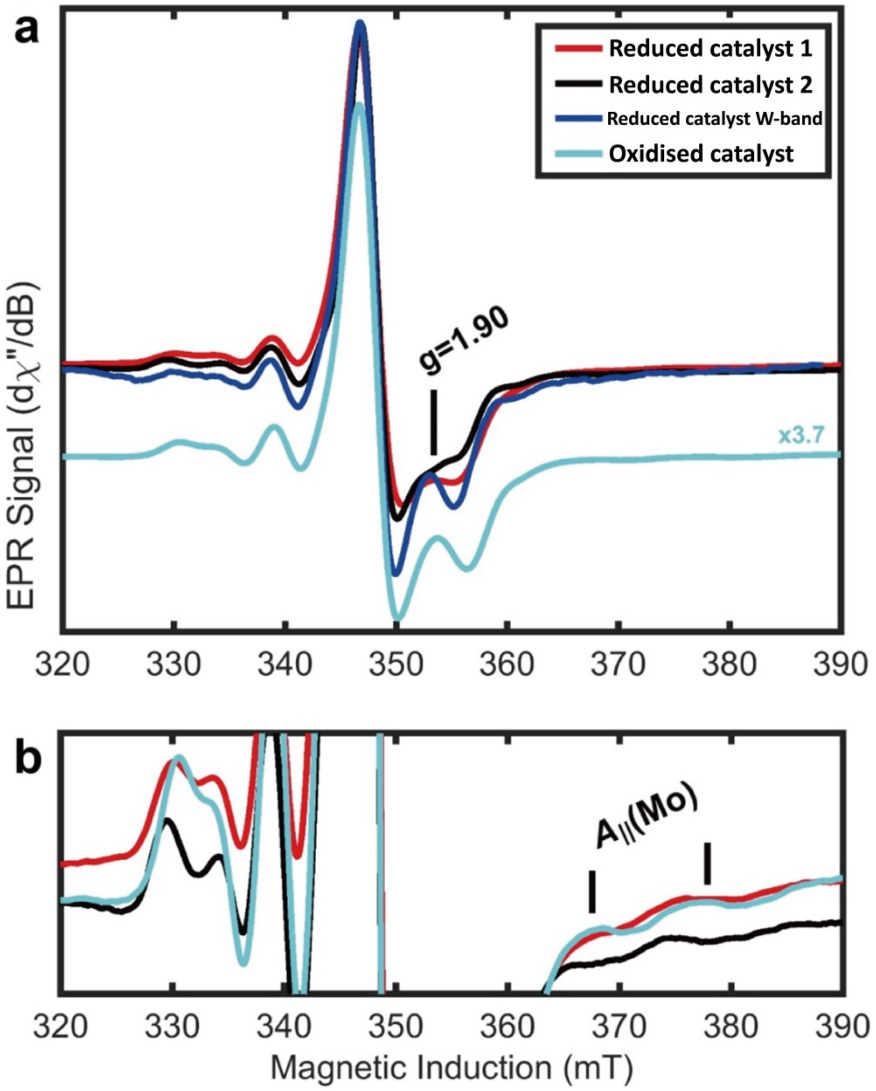

Supplementary Figure 23. X-band CW-EPR data of reduced catalyst variation in spectral shape (red, black and blue), with the oxidised catalyst in cyan. A vertical expansion of panel **a** is given as panel **b**. Two typical X-band samples of ca. 6 mg of material, but prepared at different times, are red and black, and a sample for W-band of ca. 0.5 mg, allowed to age in a capillary for six months, is shown in blue. The g=1.90 line in panel **a** and two of six $A_{\|}$(Mo) lines in panel **b** indicate spectral differences between the black and cyan traces. Black and blue traces were acquired at 85 K, while the red trace was at 10 K, all under non-saturating acquisition conditions. The microwave frequency was 9.389 GHz.

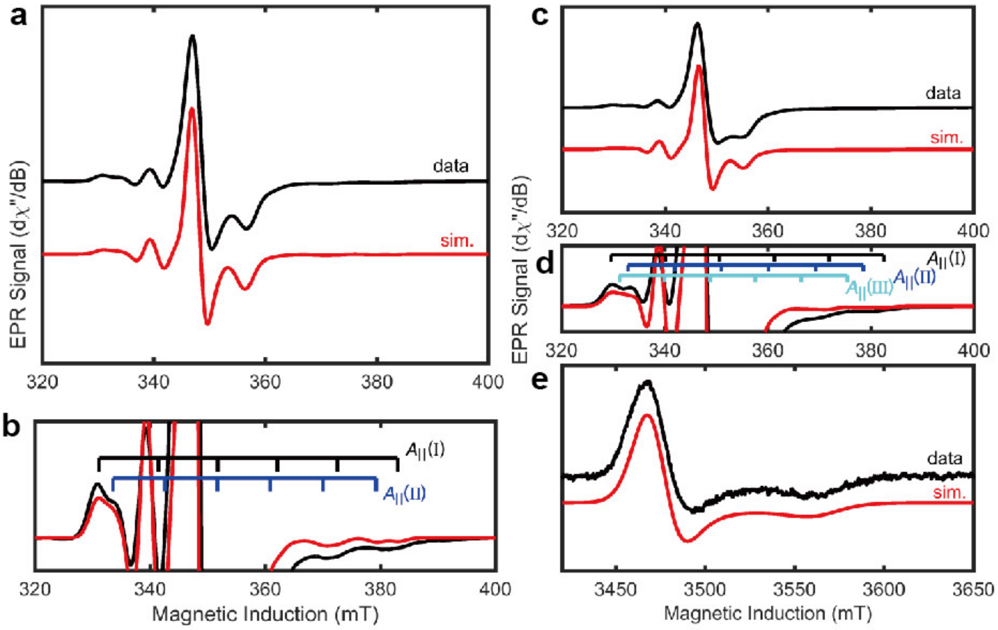

Supplementary Figure 24. X-band data and simulations of precursor, panels **a** and **b**, and X-band and W-band data and simulations of reduced catalyst, X-band in panels **c** and **d**, with W-band in panel **e**. Simulation in the precursor involves two components, by $g_{\perp}'$ = 1.935, $g_{\parallel}'$ = 1.880, $A(^{95}Mo)_{\parallel}'$ = 273 MHz and $g_{\perp}''$ = 1.929, $g_{\parallel}''$ = 1.8835, $A(^{95}Mo)_{\parallel}''$ = 241 MHz. Simulation in the reduced form involves three components: $g_{\perp}'$ = 1.932, $g_{\parallel}'$ = 1.8833, $A(^{95}Mo)_{\parallel}'$ = 279 MHz, $g_{\perp}''$ = 1.931, $g_{\parallel}''$ = 1.8853, $A(^{95}Mo)_{\parallel}'$ = 241 MHz, $g_{\perp}'''$ = 1.933 $g_{\parallel}'''$ = 1.898, and $A(^{95}Mo)_{\parallel}'''$ = 235 MHz. In all simulations, $A(^{95}Mo)_{\perp}$= 113 MHz, being less well-determined, but is consistent with a value of ~2 for $A(^{95}Mo)_{\parallel}/A(^{95}Mo)_{\perp}$ in tetragonally-distorted Mo^{5+} octahedral.[2] Instrument settings are given in the Methods.

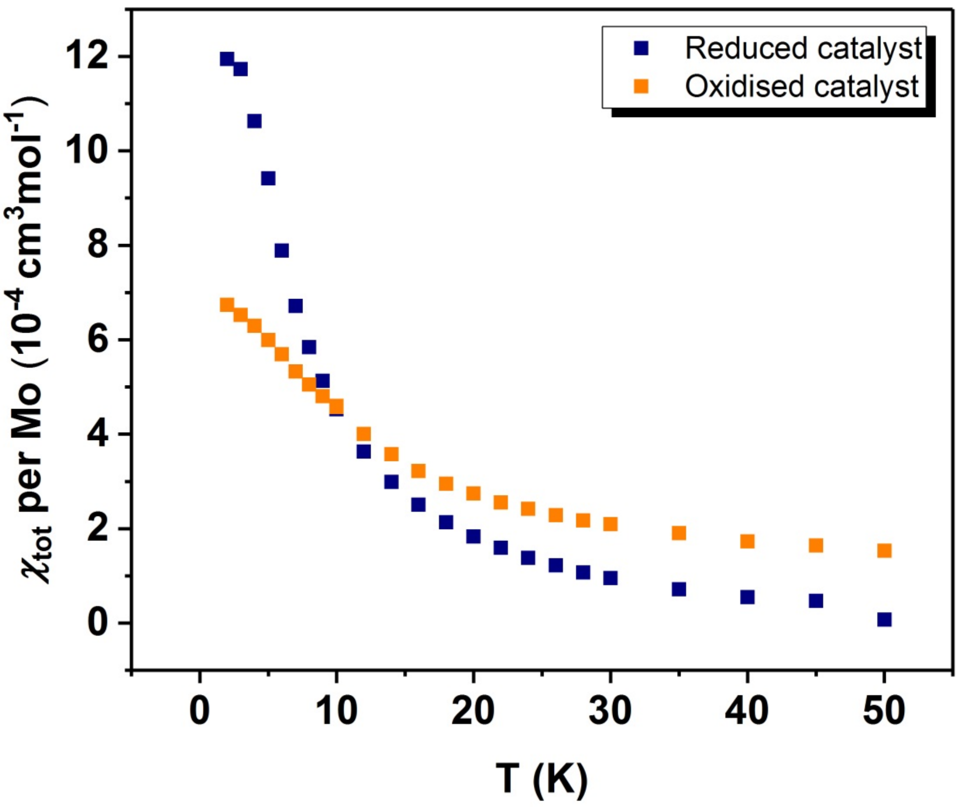

Supplementary Figure 25. Magnetic susceptibility (per Mo) at a field of 0.1 T by comparing the oxidised and reduced catalyst (yellow and blue, respectively).

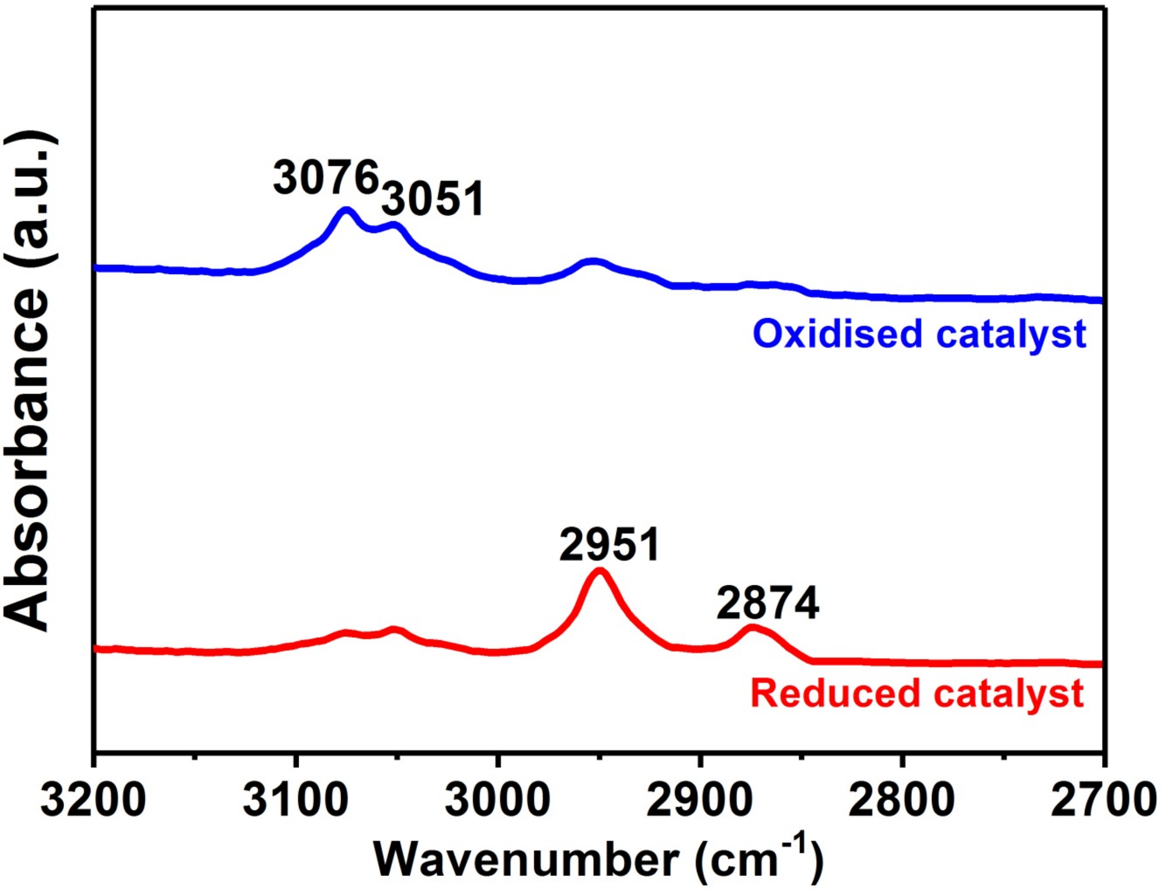

Supplementary Figure 26. DRIFTS spectra at 2700-3200 cm^{-1} obtained over oxidised catalyst and reduced catalyst (Pd/m-MoO_3-P_2O_5/SiO_2) in a reaction mixture containing phenol and He at 383 K.

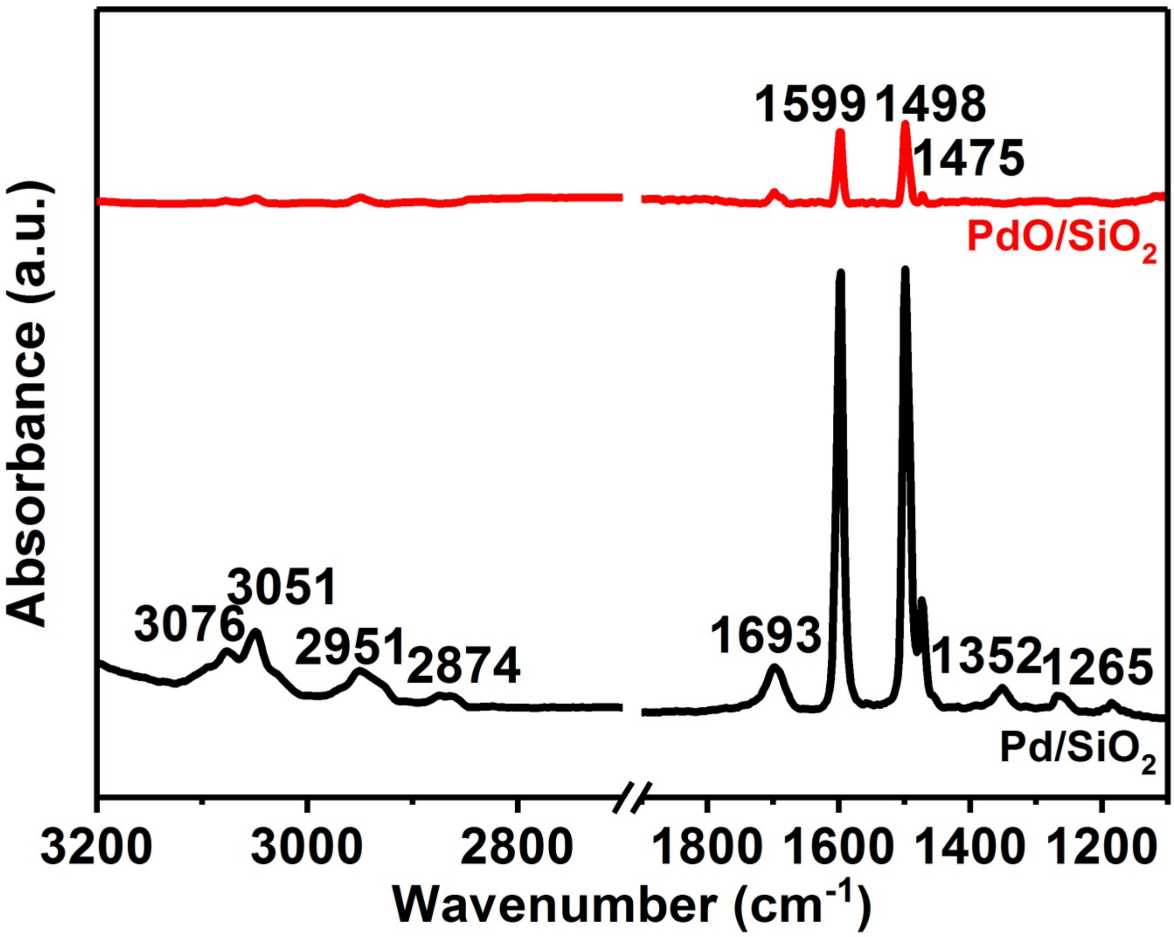

Supplementary Figure 27. DRIFTS spectra at 1100-3200 cm^{-1} obtained over oxidised Pd/SiO_2 (PdO/SiO_2) and reduced Pd/SiO_2 in a reaction mixture containing phenol and He at 383 K.

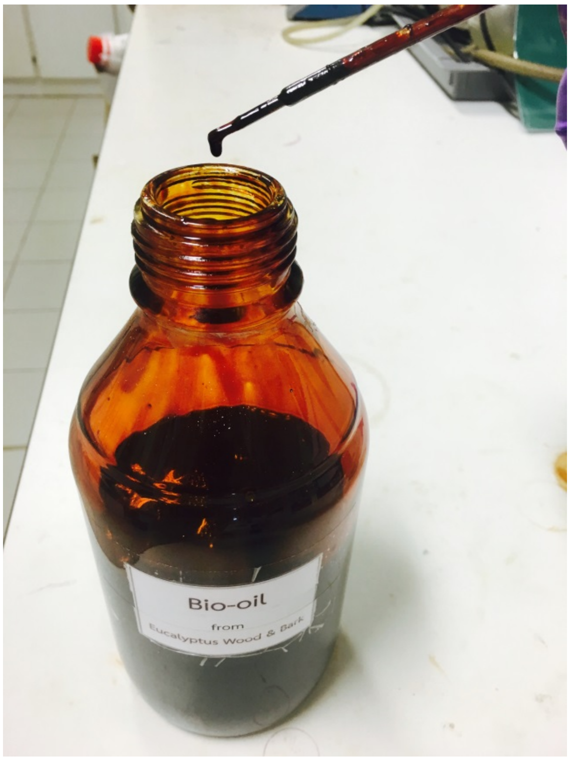

Supplementary Figure 28. Image of WIBO derived from wood and bark.

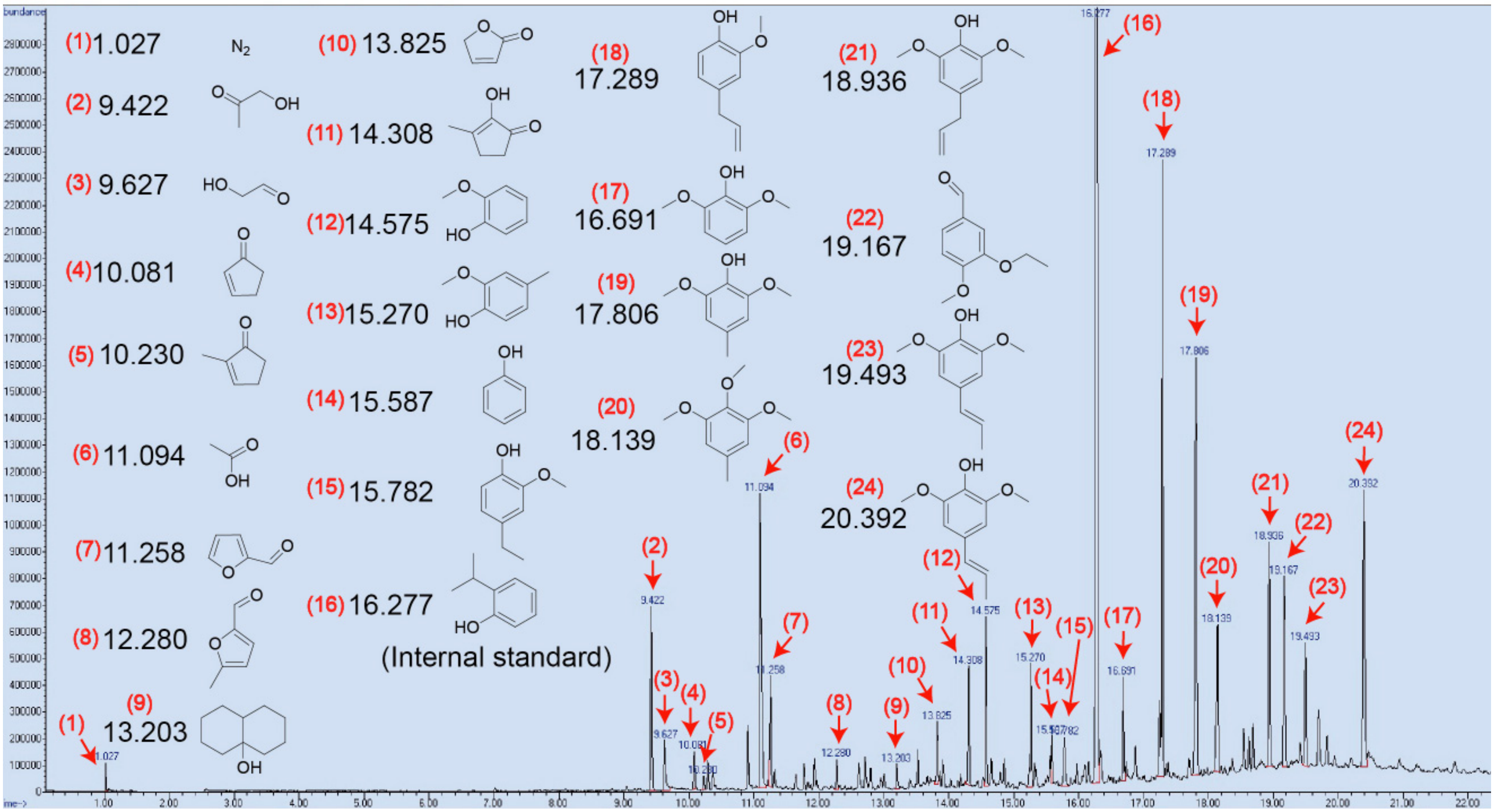

Supplementary Figure 29. Typical GC-MS spectrum of the WIBO dissolved in methanol with internal standard.

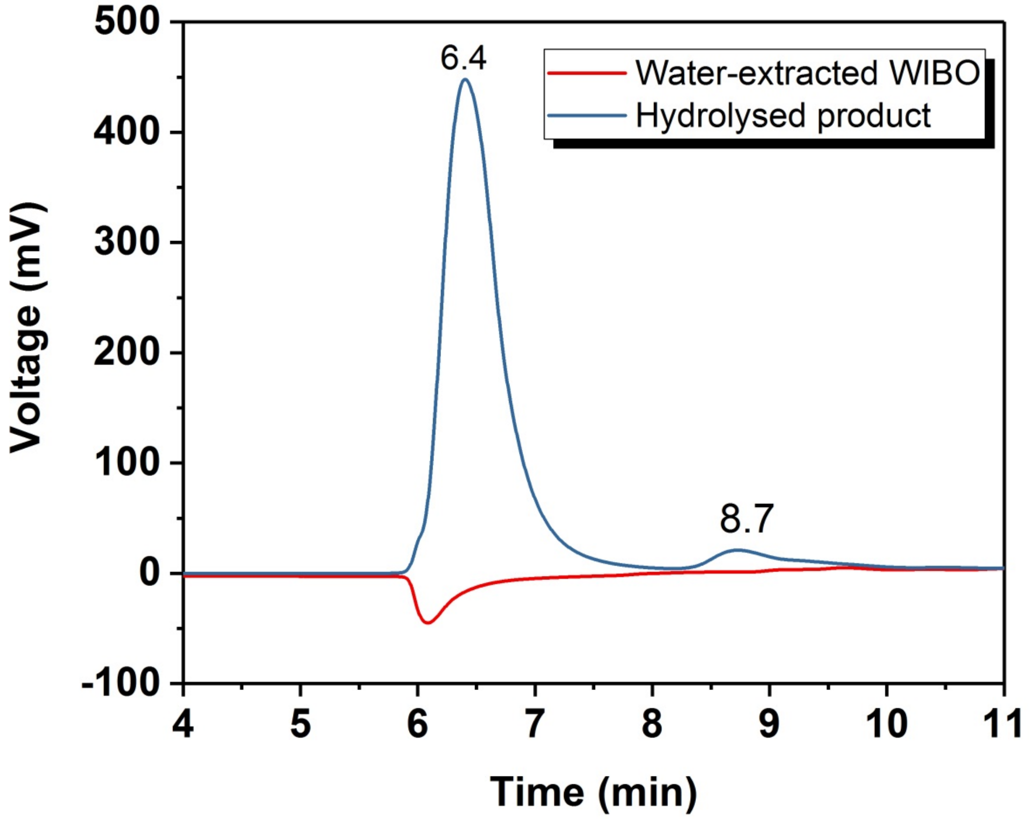

Supplementary Figure 30. HPLC spectra comparison of water extracted WIBO (red) and its hydrolysed product (dark blue). The peak shown at around 8.7 minutes corresponds to sugar monomers determined by standard glucose. The peak at 6.4 minutes originated from the acidity of the solution.

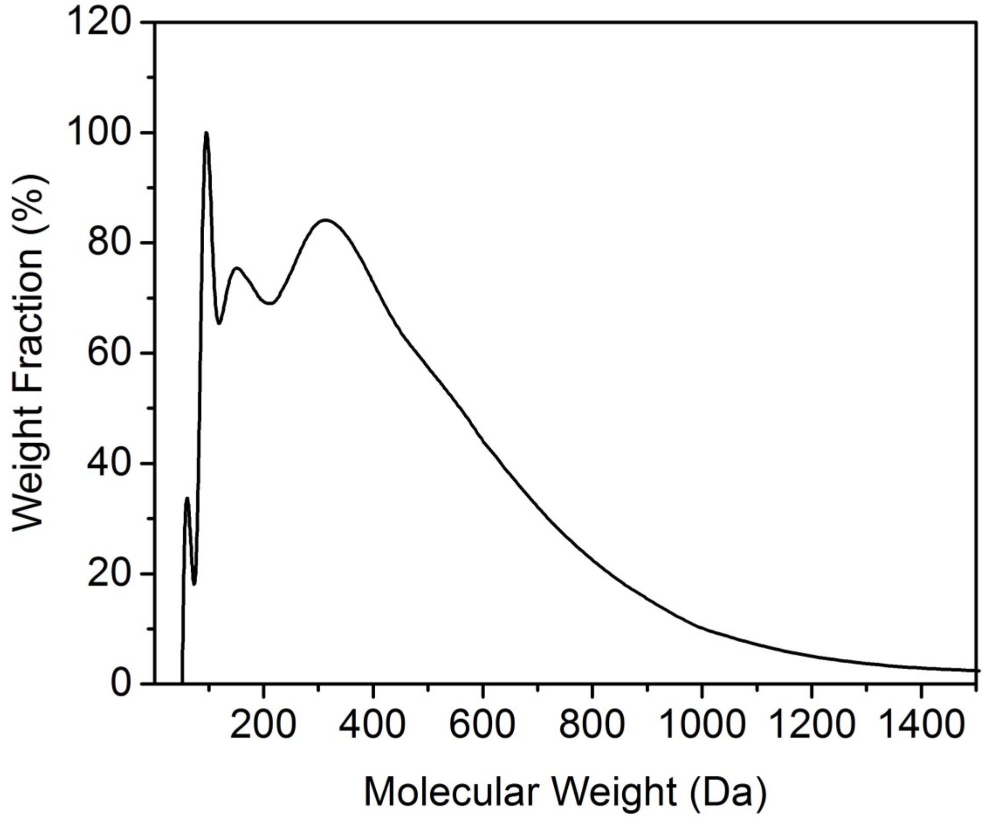

Supplementary Figure 31. Gel permeation chromatogram of WIBO.

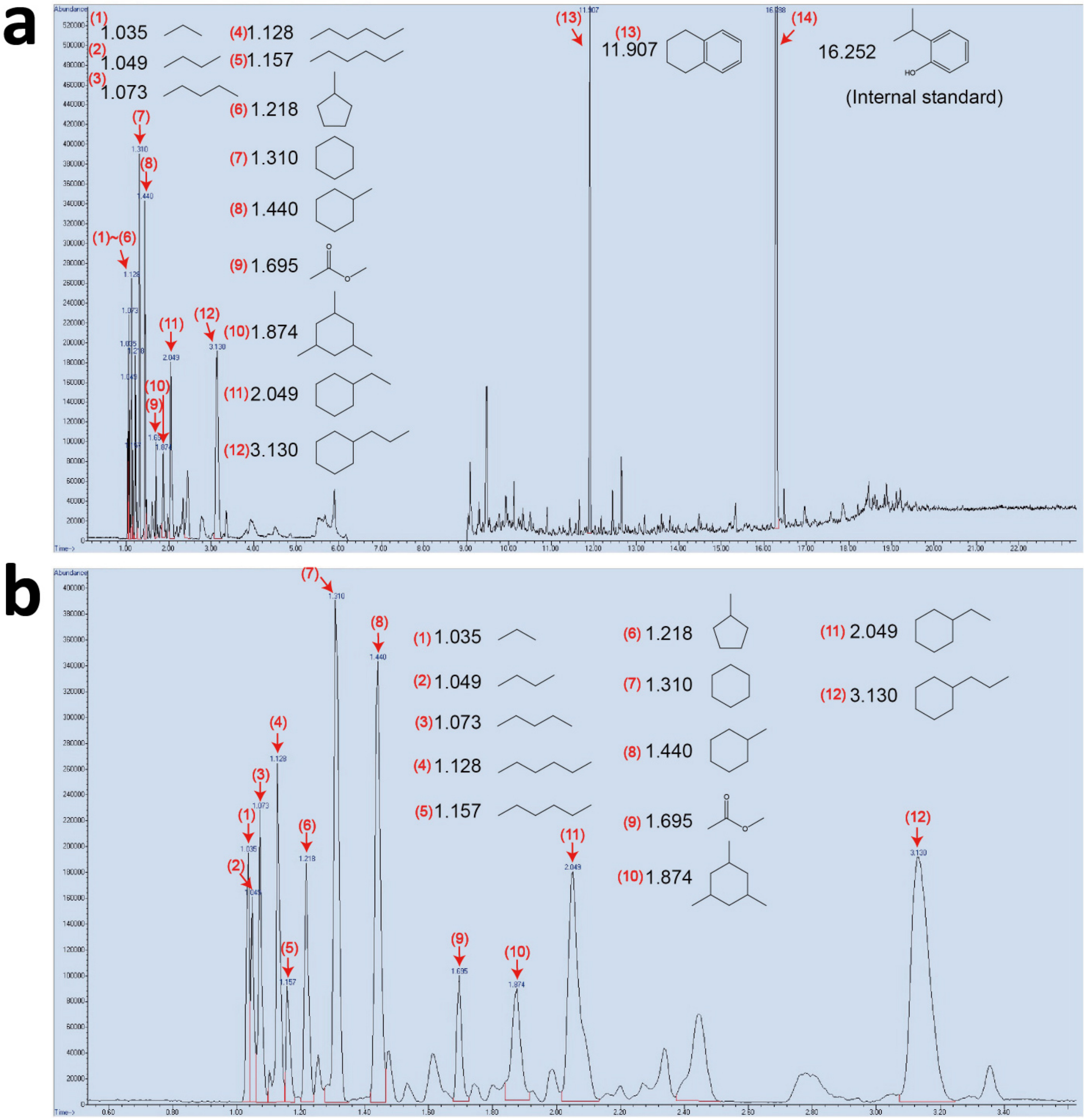

Supplementary Figure 32. **a**, Typical and **b**, enlarged region of GC-MS spectrum of the reaction effluent with internal standard obtained from hydrodeoxygenation of WIBO at 453 K, 1 MPa H_2, 4 hours, stirred at 800 rpm. MS detector was off from 6.2 to 9 minutes to block decalin (solvent). Tetralin was formed by dehydrogenation.

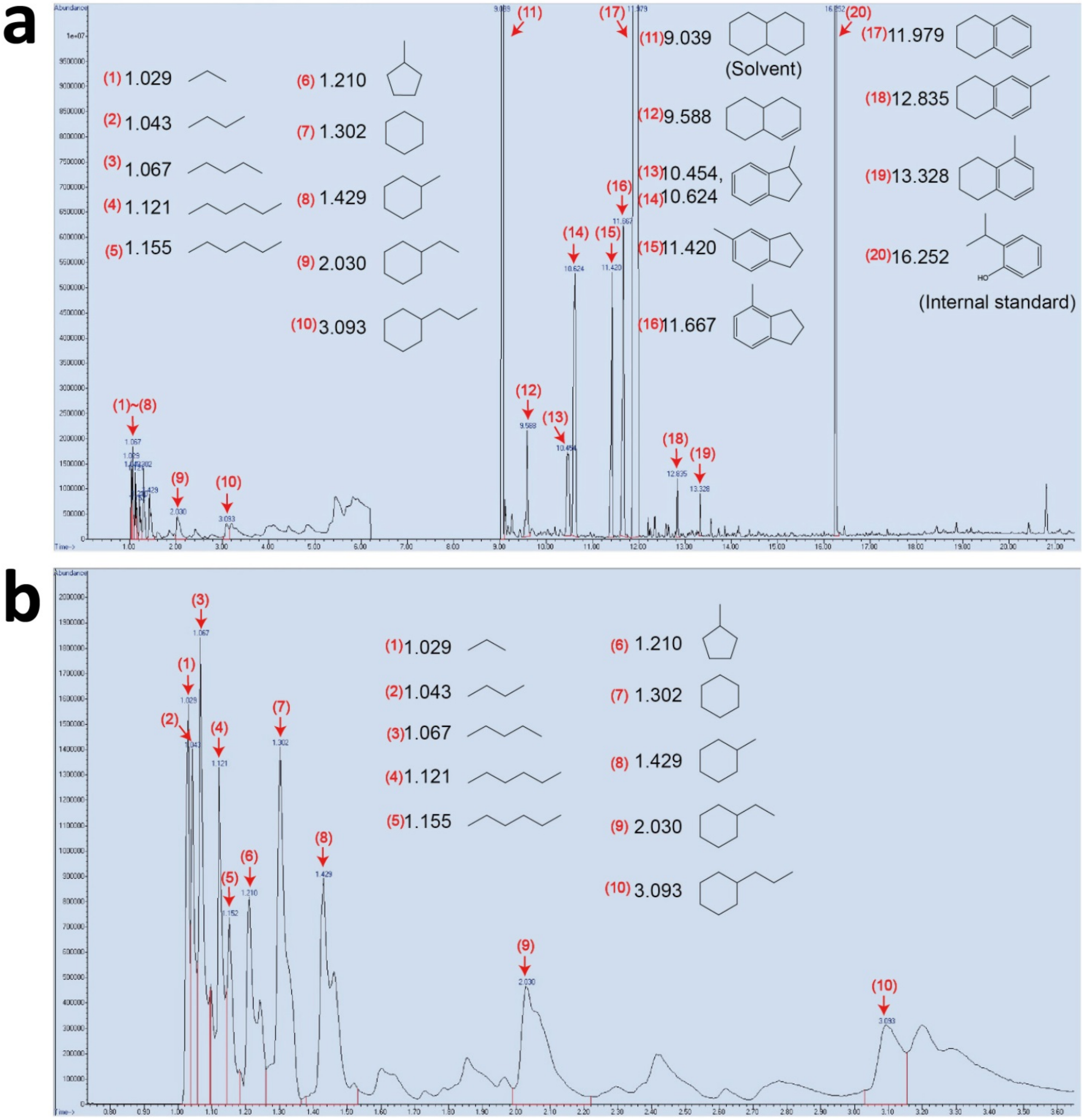

Supplementary Figure 33. **a**, Typical and **b**, enlarged region of GC-MS spectrum of the reaction effluent with internal standard obtained from hydrodeoxygenation of WIBO at 523 K, 1 MPa H_2, 15 hours, stirred at 800 rpm. MS detector was off from 6.2 to 9 minutes to block decalin (solvent). Tetralin was formed by dehydrogenation, 1-methylindane was formed by isomerisation.

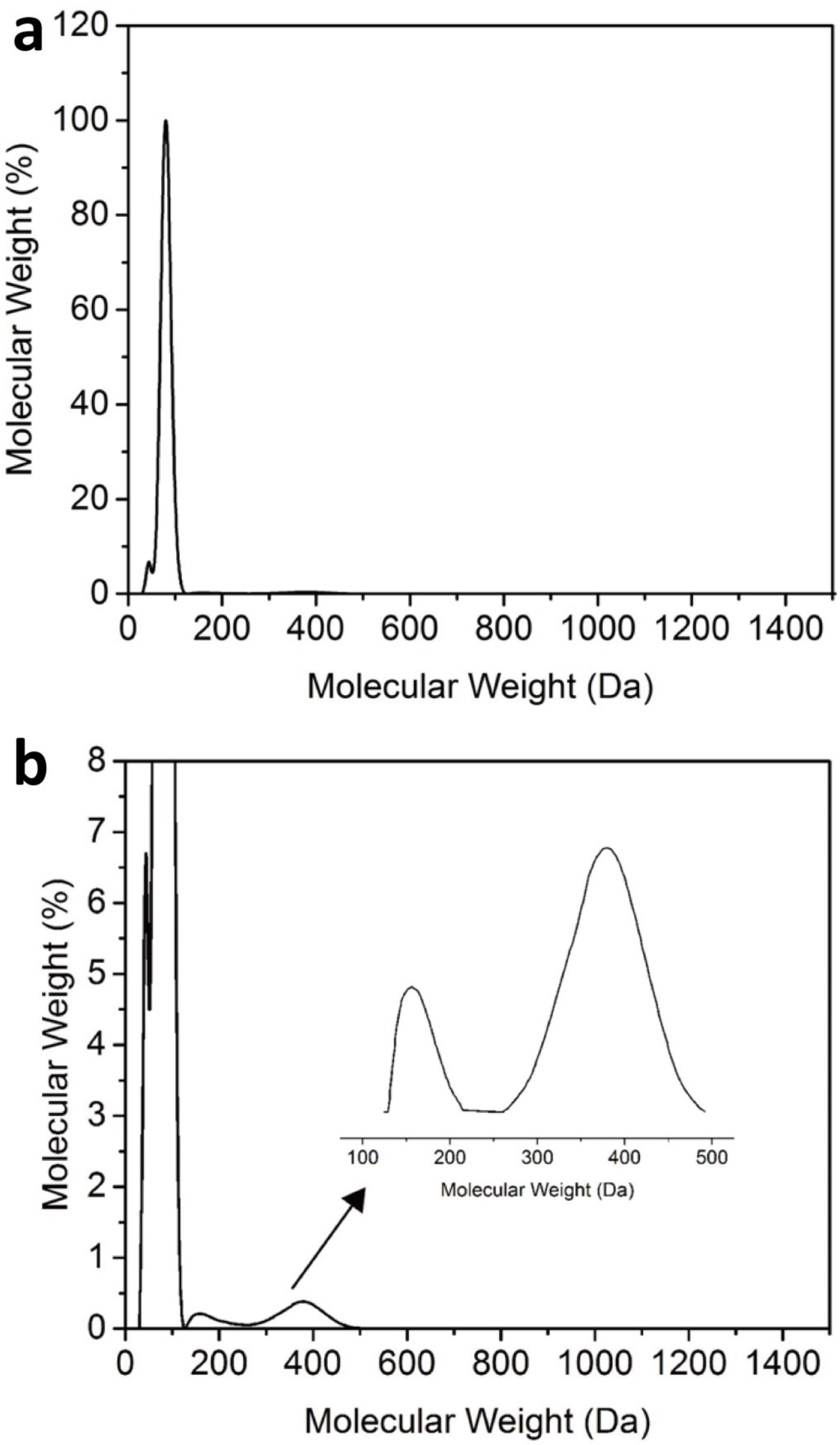

Supplementary Figure 34. **a**, Gel permeation chromatogram and **b**, enlarged region of hydrodeoxygenated WIBO. Hydrodeoxygenation carried out at 523 K, 1 MPa H_2, 4 hours, stirred at 800 rpm. Inset in b shows oligomer region from 125 – 492 Da with M_w of 302.

Supplementary Table 1. ICP-AES analysis.

**Materials**	**Pd (wt%)**	**Mo (wt%)**	**P (wt%)**
Pd/m-MoO_3-P_2O_5/SiO_2	0.48	7.7	2.2
Pd/SiO_2	0.56	-	-
Pd/P_2O_5/SiO_2	0.79	-	2.3
Spent Pd/m-MoO_3-P_2O_5/SiO_2*	0.46	6.8	2.2

*The spent sample is the one used after 419-hour continuous flow reaction and calcined at 673 K for 5 h.

Supplementary Table 2. Mo K-edge EXAFS curve Fitting Parameters[a]

			R (Å)				
sample	shell	N	XRD/DFT	EXAFS	σ^2 (Å^2)	ΔE_0 (eV)	R, %
Mo bulk[b]	Mo−Mo1	**8**	2.73	2.73			0.01
	Mo−Mo2	**6**	3.15	3.15			
MoO_2[c]	Mo−O	**6**	2.01	2.02	0.004	6.4	0.01
	Mo−Mo1	**1**	2.51	2.52	0.003		
	Mo−Mo2	**1**	3.11	3.12	0.005		
MoO_3[d]	Mo−O1	**2**	1.71	1.71	0.003	1.0	0.6
	Mo−O2	**2**	1.95	1.96	0.002		
	Mo−O3	**2**	2.28	2.27	0.006		
Pd/MoO_3-P_2O_5/SiO_2[e]	Mo−O1	2.2	−	1.72	0.002	0.5	0.6
	Mo−O2	0.9	−	1.98	0.003		
	Mo−O3	1.4	−	2.11	0.008		
	Mo−O4	0.9	−	2.33	0.006		
	Mo−Pd/Mo[f]	0.5	−	2.65	0.009		
	Mo−P/Si[f]	0.9	−	3.33	0.005		
Pd/m-MoO_3-P_2O_5/SiO_2[e]	Mo−O1	1.9	1.70	1.72	0.002	3.6	0.8
	Mo−O2	1.2	1.95	1.99	0.003		
	Mo−O3	1.5	2.03	2.13	0.008		
	Mo−O4	0.4	2.48	2.34	0.006		
	Mo−Pd/Mo[f]	0.7	2.75	2.66	0.009		
	Mo−P/Si[f]	1.1	3.39	3.37	0.005		

[a]*N*, coordination number; *R*, distance between absorber and backscatter atoms; σ^2, Debye–Waller factor to account for both thermal and structural disorders; ΔE_0, inner potential correction; *R* factor (%) indicates the goodness of the fit. Error bounds (accuracies) that characterize the structural parameters obtained by EXAFS spectroscopy were estimated as $N \pm 20\%$; $R \pm 1\%$; $\sigma^2 \pm 20\%$; $\Delta E_0 \pm 20\%$. S_0^2 was fixed to 0.9 as determined from Mo foil fitting. Bold numbers indicate fixed coordination number (*N*) according to the crystal structure. O1, O2, O3 and O4 represent the first, second, third and fouth nearest

neighbor coordination O atoms. [b]Fitting range: 3.1 ⩽ k (/Å) ⩽ 15.7 and 2.0 ⩽ R (Å) ⩽ 3.2. [c]Fitting range: 3.0 ⩽ k (/Å) ⩽ 14.9 and 1.1 ⩽ R (Å) ⩽ 3.0. [d]Fitting range: 3.8 ⩽ k (/Å) ⩽ 15.0 and 1.1 ⩽ R (Å) ⩽ 2.1. [e]Fitting range: 3.7 ⩽ k (/Å) ⩽ 15.7 and 0.8 ⩽ R (Å) ⩽ 3.3. [f]Because the P/Si atoms are indistinguishable by XAFS due to their nearly identical scattering ability of photoelectron, only Mo-P path is used; also the Mo-Pd and Mo-Mo paths are not distinguished. [g]DFT calculated values for the structural configuration shown in Figure 2h.

Supplementary Table 3. Pd K-edge EXAFS curve Fitting Parameters[a]

			R (Å)				
sample	shell	*N*	XRD/DFT	EXAFS	σ^2 (Å^2)	ΔE_0 (eV)	*R*, %
Pd bulk[b]	Pd−Pd	**12**	2.75	2.74	0.006	0.4	0.01
PdO[c]	Pd−O1	**4**	2.02	2.03	0.003	2.9	0.4
	Pd−Pd1	**4**	3.04	3.05	0.008		
	Pd−Pd2	**8**	3.42	3.43	0.014		
	Pd−O2	**8**	3.65	3.54	0.022		
Pd/MoO_3-P_2O_5/SiO_2[d]	Pd−O1	5.6	−	2.02	0.005	3.9	0.6
	Pd−Pd/Mo[f]	0.4	−	2.76	0.010		
	Pd−P/Si[f]	0.9	−	3.10	0.012		
	Pd−O2	3.2	−	3.91	0.008		
Pd/m-MoO_3-P_2O_5/SiO_2[e]	Pd−O1	4.8	1.97[g]	2.03	0.005	5.1	0.4
	Pd−Pd/Mo[f]	1.5	2.75[g]	2.80	0.010		
	Pd−P/Si[f]	3.6	3.23[g]	3.12	0.010		
	Pd−O2	7.3	3.75[g]	3.93	0.008		

[a]*N*, coordination number; *R*, distance between absorber and backscatter atoms; σ^2, Debye–Waller factor to account for both thermal and structural disorders; ΔE_0, inner potential correction; *R* factor (%) indicates the goodness of the fit. Error bounds (accuracies) that characterize the structural parameters obtained by EXAFS spectroscopy were estimated as $N \pm 20\%$; $R \pm 1\%$; $\sigma^2 \pm 20\%$; $\Delta E_0 \pm 20\%$. S_0^2 was fixed to 0.89 as determined from Mo foil fitting. Bold numbers indicate fixed coordination number (*N*) according to the crystal structure. O1 (Pd1) and O2 (Pd2) represent the first and second nearest neighbor coordination O (Pd) atoms. [b]Fitting range: $2.9 \leqslant k$ (/Å) $\leqslant 16.7$ and $2.0 \leqslant R$ (Å) $\leqslant 3.0$. [c]Fitting range: $2.9 \leqslant k$ (/Å) $\leqslant 11.8$ and $1.0 \leqslant R$ (Å) $\leqslant 3.8$. [d]Fitting range: $2.8 \leqslant k$ (/Å) $\leqslant 11.1$ and $0.9 \leqslant R$ (Å) $\leqslant 3.4$. [e]Fitting range: $2.8 \leqslant k$ (/Å) $\leqslant 11.1$ and $0.9 \leqslant R$ (Å) $\leqslant 3.7$. [f]Because the P/Si atoms are indistinguishable by XAFS due to their nearly identical scattering ability of photoelectron, only Pd-P path is used; also the Pd-Pd and Pd-Mo paths are not distinguished. [g]DFT calculated values for the structural configuration shown in Figure 2h.

Supplementary Table 4. Comparison of the bifunctional metal-acid catalysts in the hydrodeoxygenation of phenol.

Reference	Catalyst	Temperature (K)	Pressure (MPa)	Time (h)	Conversion	Selectivity of cyclohexane	Metal /substrate
[3]	Pd/C-H_3PO_4	473<br>453	5	0.5	98%<br>8.6%	92%<br>N.D.	0.0196
[4]	Pt NPs /[bmim][BF_4]<br>Rh NPs /[bmim][TF_2N]	403	4	4	48%<br>98%	98%<br>84%	0.003
[5]	Ru NPs@SILP-1.00	448	12	16	93%	95%	0.001
This work	Pd/m-MoO_3-P_2O_5/SiO_2	383	1	2	99.6%	97.2%	0.002

Supplementary Table 5. Quantitative analysis of the acidities in different samples.

Sample	BET surface area (m^2/g)	Peak									Brønsted acid density (mmol/g)	Lewis acid density (mmol/g)
		B-acid I			B-acid II			L-acid III				
		ppm	Area(%)	TMPO (mmol/g)	ppm	Area(%)	TMPO (mmol/g)	ppm	Area(%)	TMPO* (mmol/g)		
Reduced catalyst, Pd/m-MoO_3-P_2O_5/SiO_2 (P:Mo = 1:1)	291.5 ±0.4	58.0	48.3%	2.99	73.6	16.3%	1.01	45.4	35.4%	2.19	4.00 (0.0138 (mmol/m^2)	2.19 (0.0075 mmol/m^2)
Oxidised catalyst (H_2 treating precursor)	-	59.8	59.3%	1.49	75.2	40.7%	1.02	-	-		2.51	0.00
Pd/SiO_2 (P:Mo = 0:0)	-	-	-	0.00	-	-	0.00	42.9 (physical sorption)	100%	0.0979	0	0.0979
Pd/MoO_3/SiO_2 (P:Mo = 0:1)	-	58.9	36.4%	0.16	71.9	13%	0.06	44.5	50.6%	0.2298	0.22	0.23
Pd/MoO_3-P_2O_5/SiO_2 (P:Mo = 2:1)	-	60.0	44.7%	0.35	76.3	23.5%	0.19	49.8	31.8%	0.251	0.54	0.25
Pd/P_2O_5/SiO_2 (P:Mo = 1:0)	-	50.7	100%	0.33	-	-	0.00	-	-	0	0.33	0.00

Supplementary Table 6. Catalytic comparison for dehydration of cyclohexanol to cyclohexene on different catalysts with varied molar ratio of P:Mo.[a]

Samples	P:Mo	Brønsted acid density (mmol/g)	Conversion (%)
Pd/SiO_2	0:0	0.00	0.0[b]
Pd/MoO_3/SiO_2	0:1	0.22	19.8
Pd/m-MoO_3-P_2O_5/SiO_2	1:1	4.00	90.2
Pd/m-MoO_3-P_2O_5/SiO_2	2:1	0.54	25.2
Pd/P_2O_5/SiO_2	1:0	0.33	12.8[c]

[a]Reaction conditions: cyclohexanol (0.195 mmol), catalyst (10 mg), decalin (7 mL), 383 K, 1 MPa N_2, 1 hours; reaction mixture stirred at 800 rpm. [b]Pd/P_2O_5/SiO_2 sample has relatively lower dehydration activity (except Pd/SiO_2), although its Brønsted acid sites is not the lowest. This could be attributed to its weaker Brønsted acid strength shown in Supplementary Table 5.

Supplementary Table 7. Elemental analysis of WIBO.*

**Elements**	**Results**	**Units**
Carbon	47.2	% wt/wt
Hydrogen	7.3	% wt/wt
Nitrogen	0.2	% wt/wt
Oxygen	42.8	% wt/wt

*Determination of CHNO contents was performed using the ASTM D-5291 test method provided by Intertek.

Supplementary Note 1

The EXAFS analysis was not able to distinguish Mo from Pd around the absorber Pd, and this attempt was not made in our work. Our EXAFS analysis just revealed the presence of substantial Pd-Pd/Mo pairs after reduction in Pd/m-MoO_3-P_2O_5/SiO_2, which indicates the probability of both Pd-Mo and Pd-Pd pairs. For the former case, Pd is still mono-atomically dispersed; in contrast, for the latter case, the structural assignment was complicated by the probability for the mixtures of several Pd species. Nevertheless, the forms of Pd species, particularly concerning whether there exists Pd nanocluster, can still be deciphered based upon the corresponding characteristic distribution of the Pd-Pd bond distances. In particular, atomically dispersed Pd should exhibit only the more distant Pd-Pd coordination shell at 3.89 Å (before reduction) or the nearest Pd-Pd coordination shell at 2.75 Å (after reduction), whereas Pd nanocluster should be characterized by presence of both the first Pd-Pd coordination shell at 2.75 Å and the second Pd-Pd coordination shell at 3.89 Å. The latter argument can be roughly obtained from the EXAFS result for Pd bulk, where strong peaks for the first and second Pd-Pd coordination shells are exhibited at distances of ~2.55 and 3.74 Å (Supplementary Fig. 11a below, no phase correction was applied), respectively. Further evidences are required for Pd nanocluster, by considering the fact that the reduced coordination numbers for the Pd-Pd first and second shells and the possible presence of additional structural disorder in Pd nanocluster as compared to Pd bulk can smooth the EXAFS signals (Note: the EXAFS equation is expressed as following Supplementary Equation 1:

$$\chi(k) = S_0^2 \sum_j \frac{N_j \left| F_{eff}^j(\pi, k, R_j) \right|}{kR_j^2} \exp(-2R_j / \lambda(k) - 2k^2\sigma_j^2) \sin(2kR_j + \phi_j(k) + 2\delta_c(k)), \quad (1)$$

where N_j is the number of neighbors in the jth atomic shell, σ_j is the Debye-Waller parameter of the jth atomic shell to account for the broadening effect due to thermal and structural disorder). For thermal disorder, we performed EXAFS simulations for Pd nanocluster having different sizes with FEFF9.0 software, where the Pd nanocluster is approximated as spherical in shape and the temperature effect is accounted for by using the correlated Debye model (the DEBYE card in FEFF9.0 is set with temperature of 300 K and Debye-temperature of 274 K). The validity of the EXAFS simulation can be appreciated from the good agreement between the EXAFS results of the experiment and calculation for Pd bulk (Supplementary Fig. 11b). From Supplementary Fig. 11c below, it can be observed that, despite the decreased peak intensities for all Pd-Pd shells as a function of reduced sizes for Pd nanoclusters, the Pd-Pd first and second shell peaks (denoted as Pd-Pd^1 and Pd-Pd^2, respectively) are still obvious in all cases. The same results were also reported in the published experimental EXAFS results for different Pd nanoclusters even with the possible introduction of structural disorder under real synthetic condition[6-9]. Therefore, to identify the Pd species (or whether there exits Pd nanocluster) in our catalysts is to probe the possible presence of EXAFS peaks corresponding to the Pd-Pd first and second shells.

First, from the wavelet transform (WT) analysis of Pd K-edge EXAFS (Fig. 2), we can see that before reduction strong WT maximum at ~ 9.5 Å^{-1} which is associated with the Pd-Pd/Mo scatterings (Supplementary Fig. 6) is observed only at a distance of ~ 3.74 Å in Pd/MoO_3-P_2O_5/SiO_2, indicating atomic dispersion of Pd. After reduction, strong WT maxima at about 7.0 and 9.5 Å^{-1}, which can be respectively associated with the Pd-P/Si and Pd-Pd/Mo scatterings (Supplementary Fig. 6), are shown at a distance of ~ 2.55 Å surrounding Pd atoms in reduced catalyst, without apparent WT intensity distribution at distance of ~3.74 Å that corresponding to the Pd-Pd second shell peak. This is in marked contrast to the WT intensity distribution in oxidised catalyst (Fig. 2f), suggesting that the

Pd/Mo coordinators approach Pd center due to creation of oxygen vacancies after reduction and thus atomic Pd dispersion is still kept in Pd/m-MoO_3-P_2O_5/SiO_2.

Second, a reduction to absurdity can be applied to exclude the existence of Pd nanocluster. Let us suppose the existence of Pd nanocluster in our reduced catalyst (Pd/m-MoO_3-P_2O_5/SiO_2) at first. The Pd-Pd first shell bond-distance obtained by EXAFS analysis is 2.76/2.80 Å in present study. Besides, we note that the Pd-Pd bond-distance was reported to increase with reduced Pd nanocluster sizes and the Pd-Pd bond-distance was shown to be ~2.80 Å for a Pd nanocluster of ~ 3 nm in the literature.[6-9] Therefore, the Pd nanocluster in our present study should be ~ 3 nm if they exist, which is consistent with TEM results that the average size for the synthesized catalysts is ~2.8 nm. Then, based on the predicted dependence of the average Pd-Pd coordination number (CN) on the Pd nancluster sizes (Supplementary Fig. 12), the average CN(Pd-Pd) for a Pd nancluster of 3 nm should be ~10.4. Given that the Pd species in Pd/m-MoO_3-P_2O_5/SiO_2 can be considered as a mix of atomically dispersed Pd and Pd nanocluster of 3 nm and the average CN(Pd-Pd) in Pd/m-MoO_3-P_2O_5/SiO_2 obtained by EXAFS is 1.5, the relative fraction of Pd nancluster of 3 nm in total Pd atoms of the reduced catalyst should be 1.5/10.4, i.e., ~15% (Note: The average CN(Pd-Pd) was used to estimate the relative fraction of Pd engaged in Pd nanoclusters of 3 nm in the reduced sample. Since CN(Pd-Pd) for atomically dispersed Pd is 0 and CN(Pd-Pd) in Pd nanoclusters of 3 nm is 10.4, the average CN(Pd-Pd) in the reduced catalyst comprising both atomically dispersed Pd (the CN(Pd-Pd)) and Pd nanoclusters of 3 nm is a linear function of the relative fractions of both structures: $CN_{Pd\text{-}Pd} = 0\times(1-\theta) + 10.4\times\theta = 10.4\theta$, which where θ is the relative fraction of Pd engaged in Pd nanoclusters of 3 nm and $(1-\theta)$ the relative fraction engaged in atomically dispersed Pd. So, $\theta = CN_{Pd\text{-}Pd}/10.4$. Similar method was used in previous work.[10] Because the Pd has been revealed be atomically distributed in Pd/MoO_3-P_2O_5/SiO_2, the Pd K-edge EXAFS results for Pd/m-MoO_3-P_2O_5/SiO_2 can be approximated by a weighted mix of the EXAFS signals from Pd/MoO_3-P_2O_5/SiO_2 and Pd nanoclusters. Here, due to the lack of experimental EXAFS data for Pd nanoclusters, the above simulated EXAFS spectra were used. For comparison, identical weight ratio is applied to Pd nanocluster of 1, 2 and 4 nm. As shown in Supplementary Fig. 13 below, we can see that no matter the sizes of Pd nanocluster, pronounced Pd-Pd second shell peaks at ~3.72 Å are observed in all mixed spectra. This is drastically contrary to the experimental EXAFS result for Pd/m-MoO_3-P_2O_5/SiO_2 where no obvious Pd-Pd second shell peak is exhibited.

Finally, it should be emphasized that the molar ratio of 15% should be the upper limit for the amount of Pd nanocluster in Pd/m-MoO_3-P_2O_5/SiO_2 if we take account the fact that the Pd-Pd/Mo coordination number of 1.5 did not result completely from the contributions of the Pd nanoclusters. It means that the real molar ratio of Pd nanocluster should be less than 15%, leading a more pronounced Pd-Pd second shell peaks at ~3.72 Å. Therefore, the assumption for the existence of Pd nanocluster is in invalid. In another words, Pd nanocluste can be safely excluded from the Pd species in our reduced catalyst Pd/m-MoO_3-P_2O_5/SiO_2, and it only suggests substantial Pd-Pd pairs due to production of the oxygen vacancies.

Supplementary references

1 Hensley, A. J. R. *et al.* Enhanced Fe_2O_3 reducibility via surface modification with Pd: characterizing the synergy within Pd/Fe catalysts for hydrodeoxygenation reactions. *ACS Catal.* **4**, 3381-3392 (2014).

2 Low, W. Hyperfine structure and core polarization of the Mo^{5+} ($4d^1$) ion. *Phys. Lett.* **24**, 46-47 (1967).

3 Zhao, C., Kou, Y., Lemonidou, A. A., Li, X. & Lercher, J. A. Highly selective catalytic conversion of phenolic bio-oil to alkanes. *Angew. Chem. Int. Ed.* **48**, 3987-3990 (2009).

4 Yan, N., Yuan, Y., Dykeman, R., Kou, Y. & Dyson, P. J. Hydrodeoxygenation of lignin-derived phenols into alkanes by using nanoparticle catalysts combined with Bronsted acidic ionic liquids. *Angew. Chem. Int. Ed.* **49**, 5549-5553 (2010).

5 Luska, K. L., Migowski, P., El Sayed, S. & Leitner, W. Synergistic interaction within bifunctional ruthenium nanoparticle/SILP catalysts for the selective hydrodeoxygenation of phenols. *Angew. Chem. Int. Ed.* **54**, 15750-15755 (2015).

6 Lin, C.-M. *et al.* Size-dependent lattice structure of palladium studied by x-ray absorption spectroscopy. *Phys. Rev. B* **75**, 125426 (2007).

7 Di Vece, M. *et al.* Hydrogen-induced Ostwald ripening at room temperature in a Pd nanocluster film. *Phys. Rev. Lett.* **100**, 236105 (2008).

8 Srabionyan, V. V. *et al.* EXAFS study of size dependence of atomic structure in palladium nanoparticles. *J. Phys. Chem. Solids* **75**, 470-476 (2014).

9 Wang, J. *et al.* Determination of Nanoparticle Size by Measuring the Metal–Metal Bond Length: The Case of Palladium Hydride. *J. Phys. Chem. C* **119**, 854-861 (2015).

10 Zitolo, A. *et al.* Identification of catalytic sites for oxygen reduction in iron- and nitrogen-doped graphene materials. *Nat. Mater.* **14**, 937-942 (2015).
